# Choline Chloride–Urea Deep Eutectic Solvent/Cu–Mn Iminodiacetate Coordination Polymer as an Efficient Catalytic System for Synthesis of Morita–Baylis–Hillman Adducts with Antimicrobial Activity

**DOI:** 10.1021/acsomega.4c05386

**Published:** 2024-11-07

**Authors:** Rhuan Karlos Santos Mendes, Emelly Suelen de Freitas Reis Santos, Sandro Dutra de Andrade, Girlyanderson Araújo da Silva, José Lucas Ferreira Marques Galvão, José Roberto Dantas de Andrade dos Santos, Edeltrudes de Oliveira Lima, Rodolfo B. da Silva, Rodrigo Cristiano, Fausthon Fred da Silva, Claudio Gabriel Lima-Junior

**Affiliations:** † Department of Chemistry, Federal University of Paraíba, Campus I, João Pessoa, Paraíba 58051-900, Brazil; ‡ Department of Pharmaceutical Science, Federal University of Paraíba, Campus I, João Pessoa, Paraíba 58051-900, Brazil; § Postgraduate Program in Materials Science and Engineering − PPCEM, Federal University of Paraíba, Campus I, João Pessoa, Paraíba 58051-900, Brazil

## Abstract

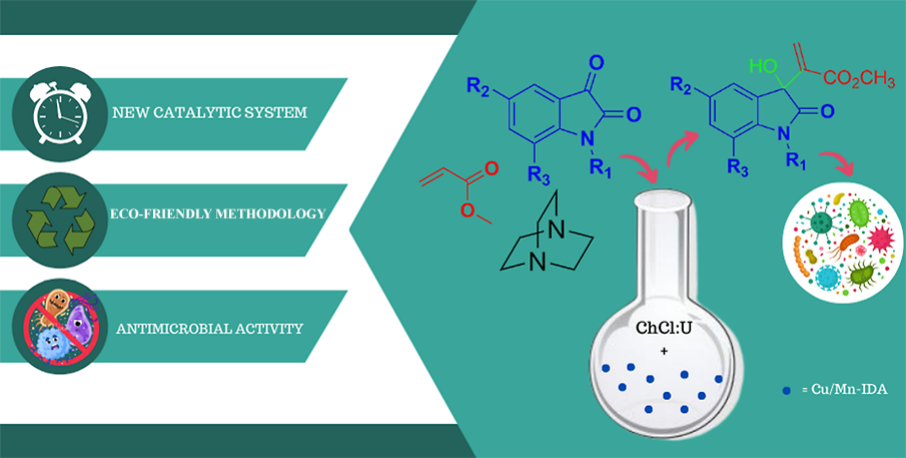

This work shows the synthesis of a series of Morita–Baylis–Hillman adducts from isatin derivatives via an efficient green approach involving the use of a new catalyst system, a mixture of copper–manganese iminodiacetate 1D coordination polymer (Cu/Mn-IDA) and choline chloride–urea deep eutectic solvent (ChCl/urea 1:2). The adducts **2a–2i** were obtained in good to excellent yields (59–97%) with shorter reaction times. The results demonstrate for the first time the synergistic catalytic effect of the combination of deep eutectic solvent and coordination polymer on the Morita–Baylis–Hillman reactions. The catalyst recyclability was investigated, and it was found that Cu/Mn-IDA maintains consistent performance for at least four catalytic cycles. The synthesized compounds were evaluated for their in vitro antimicrobial activity against four bacteria and eight fungi species. Among all, **2a**, **2b**, **2d**, **2e**, and **2g** showed antifungal and antibacterial activities in 70–83% of the strains tested. The adduct **2e** exhibited significant antifungal activity with a minimum inhibitory concentration value of 128 μg mL^–1^ compared to the standard drug fluconazole (minimum inhibitory concentration, 256 μg mL^–1^).

## Introduction

The Morita–Baylis–Hillman reaction (MBHR) is widely used in organic synthesis, involving the formation of a carbon–carbon bond between an α,β-unsaturated carbonyl (aldehyde, ketones, or imines) and a nucleophile, commonly an alkene with an electron-withdrawing group. This reaction is also typically catalyzed by a tertiary amine (R_3_N) or phosphine (R_3_P), with DABCO (1,4-diazabicyclo[2.2.2]octane) being most widely used, but always in a stoichiometric proportion.^1,2^ From the synthetic point of view, MBHR is attractive since it yields multifunctionalized products [Morita–Baylis–Hillman adducts (MBHA)], with potential biological activities. MBHAs have been studied in different pharmacological areas, such as antibacterial, antitumor, antifungal, anti-inflammatory, and antiprotozoal activities. In addition, MBHR is known to proceed in green solvents (water, for example) or even solventless, attracting attention from an ecological perspective.^3−5^

Recently, researchers have explored several variations of the MBHR to optimize experimental conditions and expand the range of acceptable substrates.^6^ In particular, the use of isatin derivatives as a substrate was first reported by independent works by Garden and Skakle and Chung et al., forming MBHA in long-term reactions.^7,8^ In these works, the authors received THF or EtOH as a solvent and a catalytic amount of DABCO (10–20 mol %). Studies on the effect of temperature and the use of DBU/CuI have also been reported for MBHR involving isatin derivatives.^9,10^

The study and focus on developing protocols to improve this reaction have been mostly addressed to solve the known inconvenient problems of MBHR, such as long reaction times and low yields. Thus, the search for new systems that increase the efficiency of catalysts is needed. In this context, recent work on MBHR has explored the use of multicatalytic systems with DABCO in the presence of coordination polymers (CPs) or deep eutectic solvents (DESs).^11−13^

CPs are metal centers bonded to organic ligands in 1D, 2D, or 3D, resulting in crystalline structures with large surface areas. These materials are largely applied in fields such as gas separation and storage, sensing, drug delivery, and as catalysts for organic reactions like epoxidation, Friedel–Crafts acylation, Knoevenagel condensation, Henry reaction, and so on.^14,15^ In 2014, CPs were applied for the first time as catalysts for MBHR. The authors reported the formation of MBHAs, obtained in high yields via a reaction between 2-cyclohexen-1-one and benzaldehyde or 2*H*-cinnamaldehyde, confirming the catalytic activity.^16^ More recently, our research group demonstrated the synthesis and characterization of three new bimetallic Cu/Mn CPs using the iminodiacetic acid ligand (named Cu/Mn-IDA). The CPs were easily obtained at room temperature, in different metals’ molar ratios.^11^ The catalytic activity of bimetallic CPs in MBHR was also investigated, using *m*-NO_2_-benzaldehyde and methyl acrylate as substrates in *N*,*N*-dimethylformamide (DMF) as solvent at room temperature, as a model reaction. No CP catalytic activity was observed in the absence of DABCO. The product was obtained in 91% yield in 5 h with Cu/Mn-IDA (0.9:0.1 ratio) as catalysts, an improved result compared to the reaction without the CP catalyst.^11^ The catalytic performance of the same ratio of Cu/Mn-IDA (0.9:0.1) CP was also similar for other substrates, with low reaction times and high yields.^12^ These results unveil the relevance of mixed-metal CPs as potential catalysts for the MBHR.

In addition, the literature also reports the use of DESs in MBHR.^13,17^ DESs have been used in several research in synthetic organic chemistry due to its easy preparation and biodegradation in the environment.^18−20^ These solvents are defined as eutectic mixtures having a melting point lower than that of the individual components.^18^ In 2016, DES was employed for the first time in MBHR between aromatic aldehydes and acrylonitrile with DABCO as a catalyst at room temperature.^18^ DES in MBHRs gave satisfactory yields, reduced reaction time, and more importantly, the possibility of reusing the solvent. Similarly, de-Andrade et al. in 2023 reported the synthesis of MBHAs from acrylonitrile- and isatin-derived substrates in DES and DABCO as a catalyst. The authors obtained the adducts in short reaction times and high yields with a significant reduction in the used amount of DABCO (50–15 mol %). The synthesized MBHAs were subjected to biological tests where one of them showed fungicidal and bactericidal activities.^13^ In this context, this work investigates the use of the catalytic properties found in CPs and DESs, jointly, aiming to improve the protocol for the synthesis of MBHAs with biological potential. The effect of the ratios of CP/DES/DABCO on the reaction results was investigated, and a discussion of the influence of this catalytic system on MBHR was presented.

## Results and Discussion

### Characterization of Catalyst Cu/Mn-10% (IDA)

**Figure 1:**
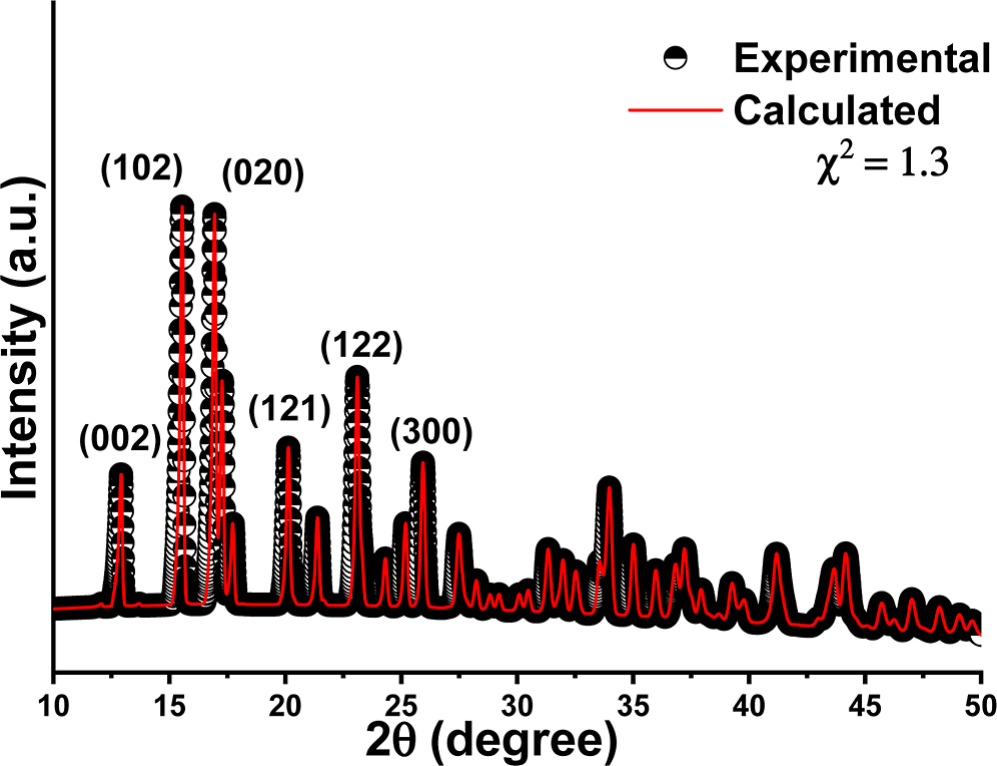
Experimental XRD pattern of the Cu/Mn-10% (IDA) catalyst.

The Cu/Mn-10% (IDA) was obtained at room temperature and characterized by powder X-ray diffraction, as shown in Figure 1. The diffraction pattern confirmed the formation of a CP isomorphous to the [Cu(IDA)(H_2_O)_2_] (Cu-IDA), due to the isomorphic substitution of Cu^2+^ ions by Mn^2+^ ions, without any significant change in the crystalline structure, as previously reported in the literature.^11,12^ Signals were indexed using the CIF file CCDC 105855, reported by Roman-Alpiste et al., and the most intense signals at 15.56 and 16.94° were related to the (102) and (020) planes. The absence of additional signals confirmed the phase purity.^21^ Cu/Mn-10% (IDA) crystallized in an orthorhombic structure and the *Pbca* space group. In this structure, each metal cation is in an octahedral environment with tetragonal distortion, coordinated by one nitrogen and five oxygen atoms (three of which are from IDA^2–^ anions and two from coordinated water molecules).^11^ Rietveld refinement was successfully used to estimate the Cu/Mn ratio, similar to reports in the literature.^11,12^ The experimental molar ratio led to the chemical formula [Cu_0.87_Mn_0.13_(IDA)(H_2_O)_2_]. This result agrees with the Mn/Cu molar ratio used in the synthetic procedure.

### Catalytic Assays

Initially, we investigated the ideal conditions for the MBHA synthesis using *N*-methylisatin (**1a**) as a substrate and methyl acrylate as a Michael acceptor. The DES ChCl/U (1:2) was selected due to satisfactory preliminary results in reactions with methyl esters. Besides, it has advantages like lower viscosity and easy manipulation compared to other DES employed in MBHR from the literature.^13^ The role of several tertiary amines [DABCO, hexamethylenetetramine (HMTA), and 1,8-diazabicyclo[5.4.0]undec-7-ene (DBU)] was also explored. For the assays, we used different molar proportions of the tertiary amine (25, 50, and 100 mol %). The amounts of catalyst CP Cu/Mn-10% (IDA) alone or in combination with 50 mol % of DABCO were 2.5, 5.0, 10.0, and 20.0 mg. Table 1 shows the results of the initial catalytic assays.

**Table 1:**
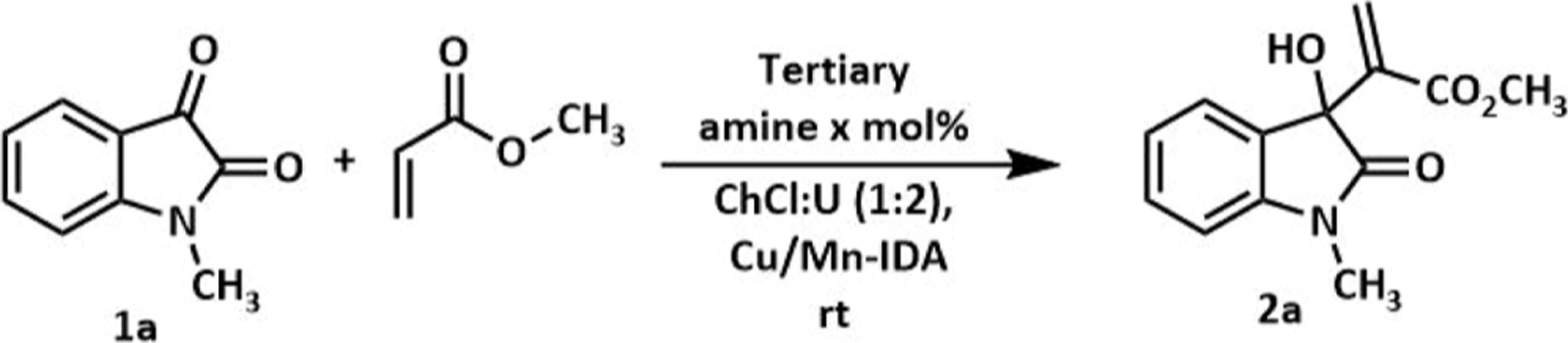
Optimization of Experimental Conditions for the Preparation of **2a**^a^

entry	catalyst mass (mg)	tertiary amine	tertiary amine (mol %)	time (h)	yield (%)^b^
1		DABCO	100	6	85
2		DABCO	50	24	88
3		DABCO	25	24	63
4		HMTA	100	24	52
5		DBU	100	24	49
6	2.5	DABCO	50	22	90^c^
7	5.0	DABCO	50	19	92^c^
8	10.0	DABCO	50	12	97^c^
9	20.0	DABCO	50	11	96^c^
10	10.0			24	NR

a Reaction conditions: 0.5 mmol *N*-methylisatin, 1.5 equiv of methyl acrylate, and 1 g of ChCl/U (1:2) under magnetic stirring at room temperature.

b Isolated yield.

c Reaction with complete consumption of the starting material.

For the MBHR illustrated in Table 1, the tertiary amines DABCO, HMTA, and DBU were initially used. When the experiments were carried out using DABCO as promoter in stoichiometric quantities, a complete reaction was observed after 6 h with an isolated yield of 85% (entry 1). DABCO has been the most used tertiary amine for this purpose; however, this catalyst has high-cost, hygroscopic characteristics, in addition to being completely lost in the aqueous phase during the product extraction. In this way, an attempt was made to reduce the reaction to catalytic quantities. At 50 mol % of DABCO (entry 2), the yield remained close to that of the previous entry. Nevertheless, the reaction was halted after 24 h due to a persistent incomplete consumption of *N*-methylisatin (**1a**). At 25 mol % of DABCO, an increase in reaction time and a considerable decrease in yield were noticed (entry 3). For HMTA and DBU (entries 4 and 5, respectively), lower reaction yields were obtained. Thus, among all tested tertiary amines, DABCO proved to be the most efficient for the studied reaction. Therefore, 50 mol % of DABCO was fixed in the following investigation of the CP catalysts’ amount.

CP catalytic investigations started using 2.5 mg of Cu/Mn-10% (IDA). With this catalytic amount (entry 6), the *N*-methylisatin (**1a**) was completely consumed in 22 h, and the MBHA was obtained in a yield of 90%, comparable to the reaction without the presence of the CP (entry 2). Higher amounts of the Cu/Mn-10% (IDA) catalysts (entries 7–9) led to a decrease in the reaction times and higher yields, with 10 mg being the optimized catalyst amount (entry 8). In addition, the reaction carried out with this optimized amount of CP and in the absence of DABCO (entry 10) gave no product even after 24 h. This result is evidence of the importance of the tertiary amine in the studied MBHR. Similar results were also observed by de Andrade et al. for other substrates in MBHR also catalyzed by CPs.^11^

**Figure 2:**
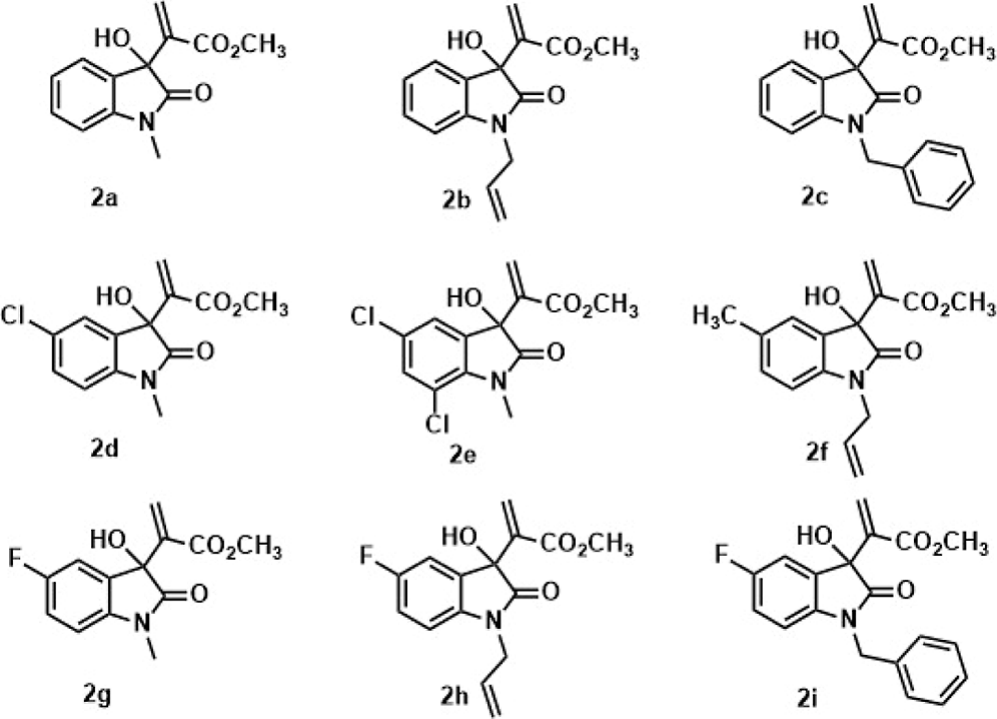
MBHAs synthesized in this work.

After establishing the best reaction conditions (entry 8), we performed MBHR with different isatin derivatives (Figure 2). *N*-Alkylated isatins were prepared by treating isatins with K_2_CO_3_ followed by methyl iodide, allyl bromide, or benzyl bromide in dry dimethylformamide (DMF) at room temperature, respectively.^13^ Results are summarized in Table 2.

**Table 2:** Times and Yields Obtained in the Preparation of **2a**–**2i**^a^

entry	MBHA	time (h)	yield (%)^b^	time/yield without CP
1	**2a**	12	97	24 h/88%^c^
2	**2b**	22	95	48 h/96%^c^
3	**2c**	19	94	36 h/98%^c^
4	**2d**	3.5	87	6 h/95%^c^
5	**2e**	3	80	5 h/75%
6	**2f**	20	58	27 h/45%
7	**2g**	16	59	20 h/40%
8	**2h**	2	81	6 h/70%
9	**2i**	3.5	62	7 h/60%

a Reaction conditions: 0.5 mmol isatin derivatives, 1.5 equiv of methyl acrylate, 10 mg of CP Cu/Mn-10% (IDA), and 1 g of ChCl/U (1:2) under magnetic stirring at room temperature.

b Isolated yield.

c See ref (13).

Isolated yields in the studied MBHRs ranged from 58 to 97%, like observed yields in the literature for these adducts in the DES/DABCO system.^9^ However, a significant decrease in the reaction time was noticed. This result is indicative of an actual cooperative effect between the Cu/Mn-10% (IDA) catalyst and DES. The solubility of substrates in the studied DES decreased with an increase in the volume of substituent bonded to the N-heteroatom, which may explain the higher reaction times in the synthesis of some adducts (entries 2 and 3). Yet, respective yields were satisfactory. Short reaction times were observed for isatin derivatives substituted with electron-withdrawing groups at positions 5 and 7 (entries 4, 5, and 7–9). These results can be related to an increase in the electrophilicity of the carbonyl carbon, making it more reactive due to the presence of electronegative atoms in the aromatic ring. However, part of these products was solubilized in the aqueous phase during isolation, decreasing the isolated yield.

**Scheme 1:**
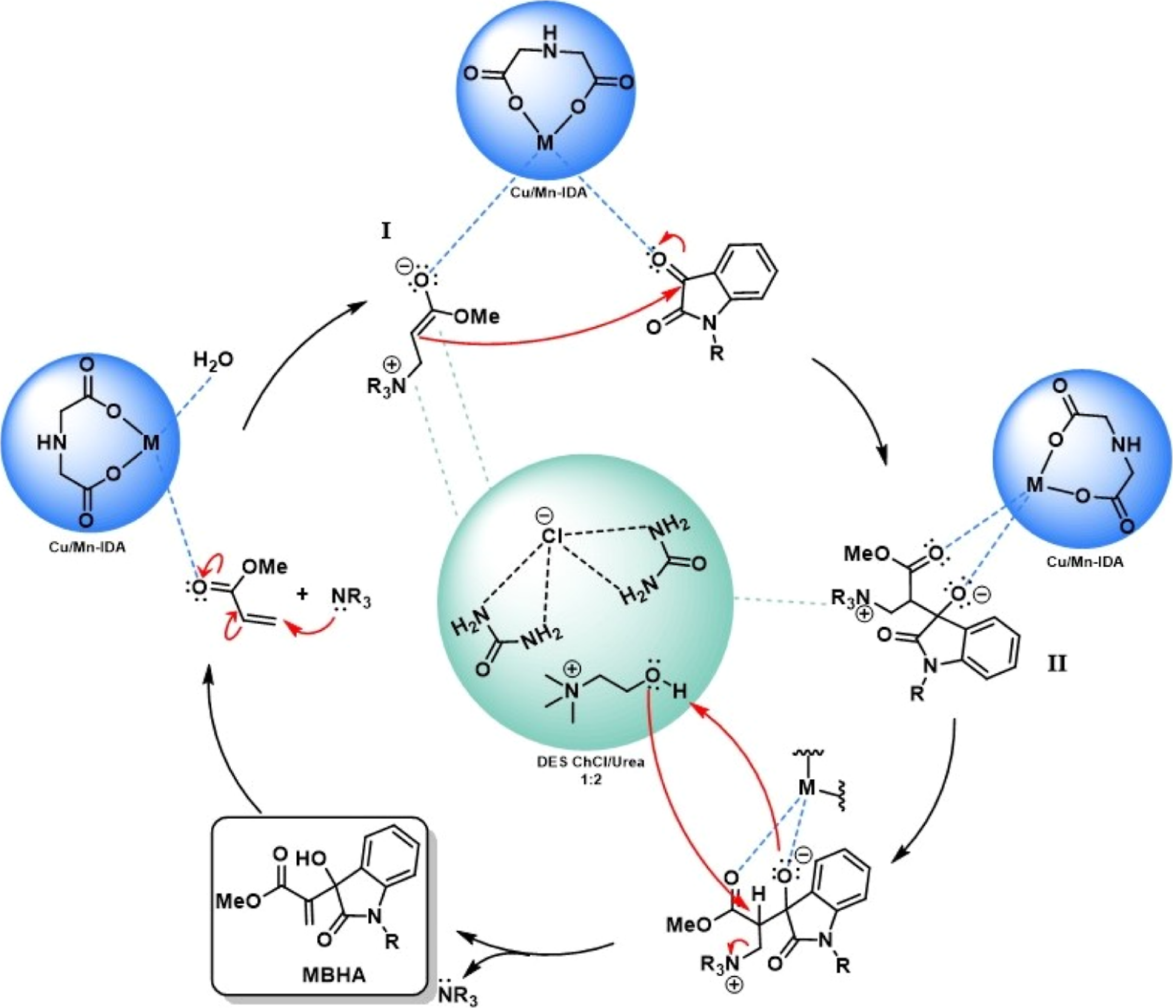
Proposed Catalytic Mechanism for the DABCO/ChCl:U/Cu/Mn-10% (IDA)

For the substrate substituted with a methyl group at position 5 of the aromatic ring (entry 6), a longer time was needed. This was an expected result since the electron-releasing effect of the methyl group diminishes the reactivity of the carbonyl group toward nucleophilic addition. For this specific reaction, 5-methyl-*N*-allyl isatin (**1f**) was not completely consumed after 20 h, and purification by a chromatographic column was necessary to remove it from the product **2f**. Recently, Kumar and Rawal developed a simple and economical method for the synthesis of isatin-derived MBHAs using a combination of CuI (10 mol %) and DBU (50 mol %) in the presence of dichloromethane as solvent. This strategy provided access to several adducts in moderate yields and a short reaction time. In this work, **2a**, **2c**, **2d**, **2g**, and **2i** were obtained in reaction times within 2–4 h and yields of 66–69%.^9^ Although our reaction times are lower than those obtained by Kumar and Rawal, the fact that we use an ecofriendly medium with a heterogeneous cocatalyst makes our results relevant.

de Andrade et al. proposed the coordination bond between the substrate and the metal center in the CP as the main aspect of the catalytic nature in these materials for MBHR.^11^ Additionally, Zhao et al. indicated the DES participating in the proton transfer step, as well as in the stabilization of MBHR intermediates.^17^ Considering these reports, here a mechanistic proposal was made (Scheme 1), starting with the activation of the carbonyls in the Michael acceptor and the isatin derivative by the CP catalyst. DABCO undergoes a 1,4 addition to the α–β-unsaturated Michael acceptor system, generating intermediate I. This zwitterionic intermediate, stabilized by DES, reacts in an aldol addition step with the isatin derivative, resulting in intermediate II. The CP continues to activate the carbonyl, making the carbon atom more electrophilic and the α-hydrogen more acidic and susceptible to being removed by DES species. Unsaturation is established with the elimination of DABCO and oxygen is also protonated by DES, generating the MBHA.

**Figure 3:**
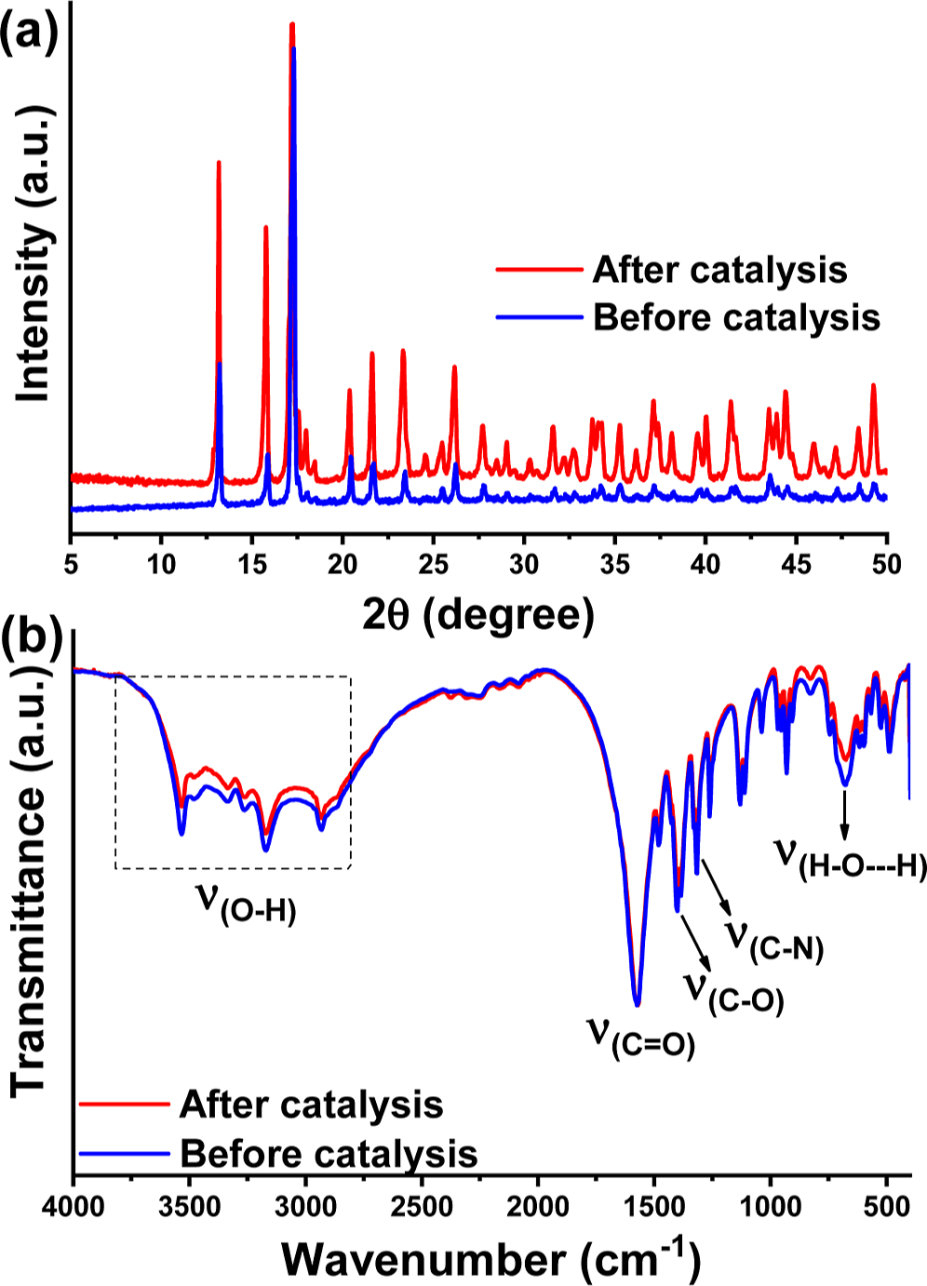
(a) XRD pattern and (b) IR spectra of the Cu/Mn-10% (IDA) before (blue lines) and after (red lines) the catalytic process.

For all MBHRs, nearly 80% of the Cu/Mn-10% (IDA) catalyst was recovered by filtration, rinsed with distilled water, dried, and kept at room temperature for further analysis. The chemical stability of the catalyst was evaluated by measuring XRD and IR spectra before and after the catalytic process (Figure 3). A high correlation between the experimental diffraction patterns of Cu/Mn-10% (IDA) (Figure 3a) before and after catalysis was observed, which indicates the total preservation of the crystalline structure of the material, showing the high stability of this catalyst under the experimental conditions investigated. Furthermore, the absence of additional signals indicates the nonformation of secondary phases. Figure 3b shows all IR signals related to the O–H, C=O, C–O, and C–N bonds of the organic groups in the iminodiacetate ligand, and the unchanged positions of these peaks after the catalytic process confirmed the maintenance of the catalyst chemical structure. Also, there may have been no incorporation of starting materials or products in the recovered catalysts because of the absence of additional signals in the IR spectrum after the catalytic process.

**Figure 4:**
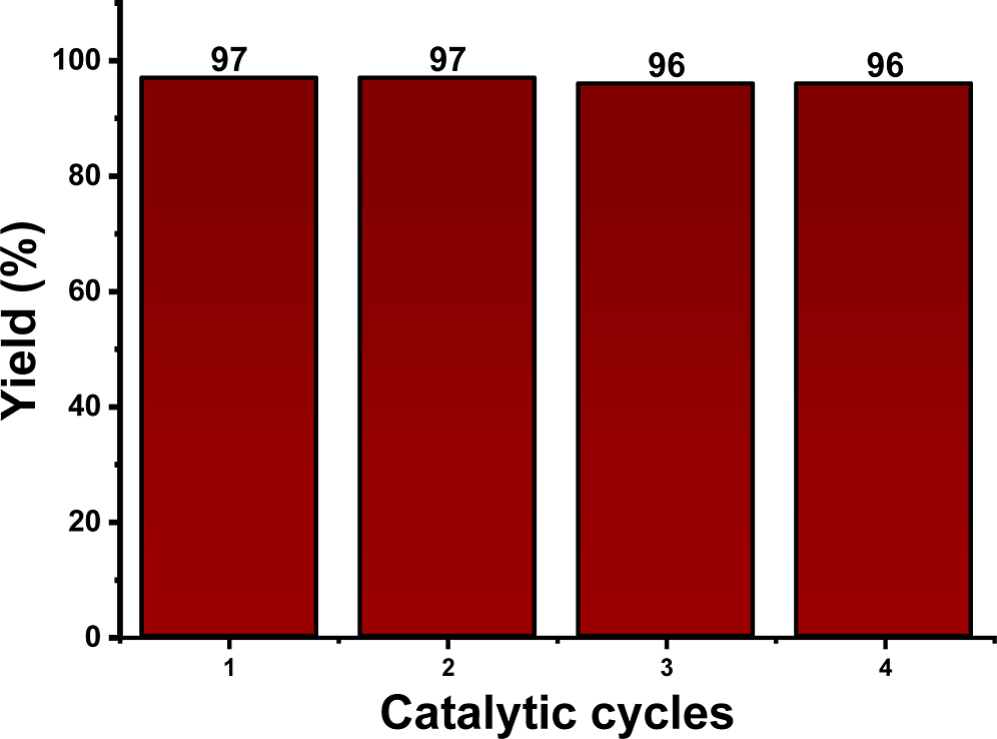
Recyclability tests for the Cu/Mn-10% (IDA) catalyst.

The recyclability of the catalyst was also evaluated using as a standard reaction for the synthesis of **2a** (Figure 4). Our results showed that the Cu/Mn-10% (IDA) catalyst could be recovered and reused for up to four reaction cycles with no significant loss in its catalytic performance. To evaluate the copper leaching, Cu/Mn-10% (IDA) was immersed under the same reaction conditions to obtain MBHA **2a** for 12 h, and the supernatant was analyzed via atomic absorption spectroscopy. A low concentration of Cu^2+^ ions was found in the reaction medium (105.36 ppm), corresponding to a loss of only 5% w/w, indicating that most of the catalyst loss occurs due to filtration processes and not due to leaching. MBHA **2a** synthesized in the presence and absence of Cu/Mn-10% (IDA) was obtained (Figure S19), and no shift in the vibrational bands was observed. Considering the metal ion affinity for polar groups, a notable presence of ions would lead to signal shifts due to coordination with carbonyl and/or hydroxyl. Similar results were found in the NMR spectra obtained here, compared to the literature.^13^ Therefore, the Cu^2+^ ion’s low leaching does not influence the purity of the obtained adducts. Additionally, the antimicrobial activity of **2a** MBHA obtained in the presence and absence of the CP is the same, proving the purity of MBHA obtained here.

### Antimicrobial Assays

Preliminary studies on the in vitro antimicrobial activity of MBHAs **2a**–**2i** were evaluated by the microdilution method with the following microbial species: *Staphylococcus aureus* ATCC-13150, Staphylococcus epidermidis ATCC-12228, *Penicillium aeruginosa* ATCC-25853, *Escherichia coli* ATCC-18739, *Candida albicans* ATCC-76485, *C. albicans* LM-70, *Candida tropicalis* ATCC-13803, *C. tropicalis* LM-77, *Aspergillus flavus* LM-26, *A. flavus* LM-55, *Aspergillus niger* LM-23, and *Penicillium citrinum* ATCC-40011 and the results are organized in Table 3.

**Table 3:** Results of the Evaluation of the MIC/MBC/MFC (1024 at 2 μg/mL) of **2a**–**2i** on Species of Bacteria and Fungi—Microdilution Technique^a^

strain	**2a**	**2b**	**2c**	**2d**	**2e**	**2f**	**2g**	**2h**	**2i**	gentamicin	fluconazole
S. aureus^b^	256	512	+	256	128	+	1024	+	+	128	NA
S. epidermidis^c^	+	512	+	256	128	+	1024	+	+	128	NA
P.aeruginosa^d^	256	512	+	256	128	+	1024	+	+	256	NA
E. coli^e^	256	512	+	256	128	+	1024	+	+	256	NA
C. albicans^f^	1024	512	+	256	128	+	1024	+	+	NA	128
C. albicans^g^	1024	512	+	256	128	+	1024	+	+	NA	256
C. tropicalis^h^	128	1024	+	256	128	+	1024	+	+	NA	256
C. tropicalis^i^	128	1024	+	256	128	+	1024	+	+	NA	256
A. flavus^j^	1024	+	+	256	128	+	1024	+	+	NA	256
A. flavus^k^	1024	+	+	256	128	+	1024	+	+	NA	256
A. niger^l^	+	+	+	+	+	+	+	+	+	NA	256
P. citrinum^m^	+	+	+	+	+	+	+	+	+	NA	256

a (+): no inhibition; NA: not applicable.

b ATCC-13150.

c ATCC-12228.

d ATCC-25853.

e ATCC-18739.

f ATCC-76485.

g LM-70.

h ATCC-13803.

i LM-77.

j LM-26.

k LM-55.

l LM-23.

m ATCC-40011.

Among the nine adducts tested, five of them (**2a**, **2b**, **2d**, **2e**, and **2g**) showed antifungal and antibacterial activities in 70–83% of the strains tested. MBHAs **2c**, **2f**, **2h**, and **2i** exhibited no effective antimicrobial activity under the tested experimental conditions. None of the investigated adducts were active against *A. niger* LM-23 and *P. citrinum* ATCC-40011. For **2a**, **2b**, **2d**, **2e**, and **2g**, the minimum inhibitory concentration (MIC) values were between 128 and 1024 μg/mL. Among them, the lowest MIC values of 256 and 128 μg/mL were obtained for **2d** and **2e**, respectively. Both of these compounds contained chloro-substituent in the aromatic ring. Adding chlorine atoms in the aromatic portion increases the lipophilicity, making **2e** more potent than reference drugs for most strains of bacteria and fungi. However, the higher antimicrobial potential of **2e** is not solely due to the increased lipophilicity. This aspect is better observed when comparing **2e** and **2i**. Adduct **2i** proved to be inactive, although it has a higher lipophilicity than **2e**. These findings may indicate that there must be interactions involving the two chlorine atoms with some specific target residue.^22,23^ Bhat and Singh synthesized MBHAs from different aromatic aldehydes and activated vinyl derivatives and evaluated their antimicrobial activity. Many of these molecules have shown potent antibacterial and antifungal activities. The *p*-chloro and *o*,*p*-dichloroaryl substituents improved the potential for antibacterial and/or antifungal bioactivities,^24^ requiring further studies on the mechanism of action for these adducts. Additionally, the minimum bactericidal concentration (MBC) and minimum fungicidal concentration (MFC) for **2d** were 1024 μg/mL, while for **2e** they were 256 to 512 μg/mL, respectively. Thus, these molecules can be considered bacteriostatic/bactericidal and fungistatic/fungicide. No tests were carried out to determine MBC and MFC for adducts **2a**, **2b**, and **2g**, as these compounds presented a high MIC value (128 to 1024 μg/mL).

## Conclusions

A series of MBHAs (**2a**–**2i**) derived from isatin was synthesized using a new catalytic system formed by choline chloride/urea (1:2) and copper–manganese iminodiacetate 1D CP (Cu/Mn-IDA). The adducts were obtained in good to excellent yields, using DABCO in a catalytic amount (50 mol %). The results demonstrate a significant decrease in the reaction time, confirming the synergistic effect between DES and CP. The CP was reused for four cycles, without losing efficiency. The adducts were subjected to biological tests to evaluate the antimicrobial activity on bacteria strains, yeast fungi, and filamentous fungi. **2a**, **2b**, **2d**, **2e**, and **2g** showed antifungal and antibacterial activities in 70–83% of the strains tested. The lowest MIC values were obtained for **2d** and **2e**. The adduct **2e** exhibited significant antifungal activity with a MIC value of 128 μg mL^–1^ compared to that of the standard drug fluconazole (MIC, 256 μg mL^–1^).

## Experimental Section

### Materials

All commercially available reagents were purchased from Aldrich and used without further purification. Reactions were monitored by TLC using Silica gel 60 UV254 Macherey-Nagel precoated silica gel plates. Flash column chromatography was performed on silica gel (300–400 mesh) using an ethyl acetate–hexane mixture as an eluent. Organic phases were dried using anhydrous Na_2_SO_4_ before evaporation on a rotary evaporator. ^1^H and ^13^C NMR spectra were recorded using a Varian Mercury Spectra AC 20 spectrometer (400 or 200 MHz for ^1^H and 100 or 50 MHz for ^13^C) using DMSO-*d*_6_ as solvent.

### Preparation of Deep Eutectic Solvent

A mixture of choline chloride (1 equiv) with urea (2 equiv) was heated at 80–100 °C for 2 h. A colorless and homogeneous liquid was then obtained, according to the literature procedure.^19^

### Preparation of CP Cu/Mn-10% (IDA)

Separately, 180 mg (0.9 mmol) of copper II acetate monohydrate and 17 mg (0.1 mmol) of manganese II sulfate monohydrate were dissolved in 5 mL of distilled water each. The solutions were mixed, and a 10 mL solution containing 270 mg (2 mmol) of the iminodiacetic acid ligand was added. The resulting mixture was homogenized and left at room temperature until blue crystals were formed. The supernatant liquid was removed, and the crystals were filtered, rinsed with distilled water, and air-dried at room temperature.^11^

### Catalytic Investigations for the Synthesis of **2a**

*N*-Methylisatin (0.5 mmol) was solubilized in ChCl/U (1:2, 1 g) under magnetic stirring, followed by the addition of methyl acrylate (1.5 mmol) and tertiary amines DABCO, HMTA, and DBU (×mol %). The reaction mixture was left at room temperature under magnetic stirring and monitored by TLC using UV light. After 24 h, **2a** was isolated by liquid–liquid extraction using 40 mL of water/ethyl acetate (1:1 v/v). Sodium sulfate anhydrous was added to remove the residual water from the organic phase, and the product was obtained by rotoevaporation. In some cases, it was necessary to perform silica gel filtration to remove residual *N*-methylisatin. This procedure was repeated adding different amounts of Cu/Mn-IDA as cocatalyst (2.5, 5, 10, and 20 mg). After the reaction time, the CP catalyst was filtered off. The resulting solution was partitioned by adding 15 mL of ethyl acetate and 15 mL of water. The organic layer was dried with Na_2_SO_4_, and the solvent was removed under reduced pressure. The respective NMR signals and the purity of **2a** have been confirmed by other previous work by our research group (see the Supporting Information).^13,25^

### General Procedure for the Synthesis of MBHAs **2b–2i**

The corresponding isatin derivatives (0.5 mmol) were solubilized in ChCl/U DES (1:2, 1 g) under magnetic stirring, followed by the addition of methyl acrylate (1.5 mmol), DABCO (50 mmol %), and the Cu/Mn-10% (IDA) catalyst (10 mg). After the reaction time, the CP catalyst was filtered off. The resulting solution was partitioned by adding 15 mL of ethyl acetate and 15 mL of water. The organic layer was dried with Na_2_SO_4_, and the solvent was removed under reduced pressure. The respective NMR signals and the purity of the products have been confirmed by other previous work (see the Supporting Information).^9,13,25^

### Characterizations

XRD data were acquired in a Shimadzu XRD-6000 diffractometer with a copper X-ray source (Kα(Cu) = 1.5481 Å) between 5 and 50° and at a scan rate of 0.01°/s. Atomic positions and Cu/Mn ratios were determined by Rietveld refinement using the software package Materials Analysis Using Diffraction. Vibrational FT-IR spectra were performed in a Shimadzu IR Prestige-21 spectrophotometer between 400 and 4000 cm^–1^, using KBr pellets. The copper ion leaching process was investigated using an atomic absorption spectrometer AA-6300, equipped with a graphite furnace (GFA-EX7i model) and an autosampler (ASC-6100 model).

### Antibacterial Assay

#### Evaluate Substances

The MBHAs were subjected to biological tests to evaluate the antimicrobial activity on bacteria strains, yeast fungi, and filamentous fungi. The antifungals used as controls were fluconazole and amphotericin B (Sigma-Aldrich). The adducts were weighed and duly solubilized in 150 μL (3%) of dimethyl sulfoxide (DMSO) and 100 μL (2%) of Tween 80 was added, completing the final volume with sterilized distilled water q.s.p. to 5 mL. In this way, the initial concentration of the products was obtained at 1024 μg/mL and serially diluted to 4 μg/mL.

#### Culture Media and Microorganisms

The culture media used in the assays to evaluate biological activity were Brain Heart Infusion (BHI) and Agar Sabouraud Dextrose (ASD) purchased from Difco Laboratories Ltd., USA, France, for maintenance, respectively, of bacterial and fungal strains. For biological activity assays, BHI broth was used for bacteria and RPMI 1640 medium with l-glutamine and without bicarbonate for fungi (Difco Laboratories Ltd., USA, France and INLAB, São Paulo, Brazil). All media were prepared according to the manufacturer’s descriptions.

For the biological activity assays of tested products, the following microbial species were used: *S. aureus* ATCC-13150, S. epidermidis ATCC-12228, *P. aeruginosa* ATCC-25853, *E. coli* ATCC-18739, *C. albicans* ATCC-76485, *C. albicans* LM-70, *C. tropicalis* ATCC-13803, *C. tropicalis* LM-77, *A. flavus* LM-26, *A. flavus* LM-55, *A. niger* LM-23, and *P. citrinum* ATCC-40011. Fungal species were kept in ASD at 4 °C. Recent subcultures were used for the tests in inclined ASD (15 × 150 mm test tubes) and incubated at 35 ± 2 °C for yeasts and at room temperature (28–30 °C) for filamentous fungi. To prepare the inoculum, colonies obtained from cultures of bacterial strains in BHI medium and fungi in ASD medium were suspended in a sterile 0.9% physiological solution and adjusted according to the 0.5 tube on the McFarland standard scale to obtain 106 CFU/mL.^26−29^

#### Determination of the MIC and MFC

Antimicrobial activity assays were carried out according to the protocols of Cleeland and Squires (1991), Eloff (1998), and CLSI (2008).^23,26^ The MIC determination on bacterial and fungal strains was carried out using the broth microdilution technique with a cell culture plate (TPP/Switzerland/Europa) containing 96 wells with a “U” bottom. Initially, 100 μL of a double-concentrated RPMI 1640 broth was distributed into the wells of the microdilution plates. Then, 100 μL of the solubilized molecules were dispensed into the wells in the first row of the plate, and through a serial dilution at a ratio of two, concentrations of 1024 up to 4 μg/mL were obtained. Finally, 10 μL of the fungal species suspensions were added to the wells, where each column of the plate specifically refers to a species. At the same time, controls were carried out: microorganisms (RPMI + yeast) to prove the viability of the strains, culture medium (RPMI) to prove sterility, and control with amphotericin B to prove fungi inhibition. The prepared plates were aseptically closed and subjected to incubation at a temperature of 35 ± 2 °C for 24–48 h for yeast assays and at room temperature (28–30 °C)/5–7 days for filamentous fungi. The product was considered active when it inhibited at least 50% of the microorganisms used in biological activity tests^30^ and the MIC was considered and interpreted as active or inactive, according to the following criteria: up to 600 μg/mL = strong activity; 600–1500 μg/mL = moderate activity; and >above 1500 μg/mL = weak activity or inactive product.^31−33^

After reading the MIC, the test was carried out to determine the MFC. Aliquots of 10 μL of the supernatant from the wells where complete inhibition of fungal growth was observed (MIC, MIC × 2, and MIC × 4) were added to 100 μL of RPMI broth contained in new microdilution plates/96 wells and incubated for 24–48 h at 35 ± 2 °C/yeasts and at room temperature (28–30 °C)/5–7days for filamentous fungi. The tests were carried out in triplicate and the result was expressed as the arithmetic mean of the MFCs obtained in the three tests.^34,35^ At the same time, sterility control (RPMI 1640 broth) and viability of fungal species (RPMI 1640 plus the inoculum of each microorganism) were carried out.

The mode of action of the test substance was also evaluated using the MFC/MIC ratio, a methodology used by Hafidh and co-workers to specify the nature of the antimicrobial effect, considered as a fungicide when the MFC/MIC ratio is between 1:1 and 2:1. On the other hand, if the ratio is greater than 2:1, the mode of action is more likely to be fungistatic.^30^

## Supporting Information Available

^13^C NMR, ^1^H NMR, and FTIR spectra of the MBHAs (PDF)
