# Supplementary material for: Choline Chloride–Urea Deep Eutectic Solvent/Cu–Mn Iminodiacetate Coordination Polymer as an Efficient Catalytic System for Synthesis of Morita–Baylis–Hillman Adducts with Antimicrobial Activity
Source: ACS Omega. 2024 Nov 7;9(46):45911–9. doi: 10.1021/acsomega.4c05386 (PMC11579785; doi:10.1021/acsomega.4c05386)
Supplement: Supplementary file 1 — ao4c05386_si_001.pdf [file ao4c05386_si_001.pdf]

Supporting information

Choline chloride-Urea deep eutectic solvent/Cu-Mn iminodiacetate coordination polymer as an efficient catalytic system for synthesis of Morita-Baylis-Hillman adducts with antimicrobial activity.

Rhuan Karlos Santos Mendes,[a] Emelly Suelen de Freitas Reis Santos,[a] Sandro Dutra de Andrade,[a] Girlyanderson Araújo da Silva,[a] José Lucas Ferreira Marques Galvão,[b] José Roberto Dantas de Andrade dos Santos,[b] Edeltrudes de Oliveira Lima,[b] Rodolfo B. da Silva,[c] Rodrigo Cristiano,[a] Fausthon Fred da Silva,[a*] Claudio Gabriel Lima-Junior*[a]

[a] Department of Chemistry, Federal University of Paraíba, Campus I, João Pessoa – PB, Brazil.

[b] Department of Pharmaceutical Science, Federal University of Paraíba, Campus I, João Pessoa – PB, Brazil.

[c] Postgraduate Program in Materials Science and Engineering – PPCEM, Federal University of Paraíba, Campus I, João Pessoa–PB, Brazil.

* Correspondence:

Corresponding Author: claudio@quimica.ufpb.br and fausthon@quimica.ufpb.br

Table of Contents

Spectroscopy data..3

^{1}H and ^{13}C NMR spectra of 2a-2i..7

Spectroscopy data

Methyl 2-(3-hydroxy-1-methyl-2-oxoindolin-3-yl)acrylate (2a).

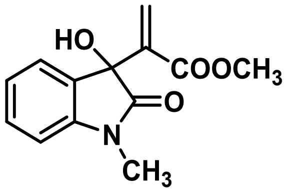

^{1}H NMR (200 MHz, DMSO-*d6*): δ 3.13 (*s*, 3H), 3.48 (*s*, 3H), 6.44 (*d*, *J* = 1,2 Hz, 1H), 6.46 (*d*, *J* = 1,2 Hz, 1H), 6.93–7.04 (*m*, 3H), 7.30 (*m*, 1H). ^{13}C NMR (50 MHz, DMSO-*d6*): δ 31.2, 56.9, 80.1, 113.7, 127.2, 128.1, 132.5, 134.7; 136.0, 144.9, 149.7, 169.6, 180.6.

Methyl 2-(1-allyl-3-hydroxy-2-oxoindolin-3-yl)acrylate (2b):

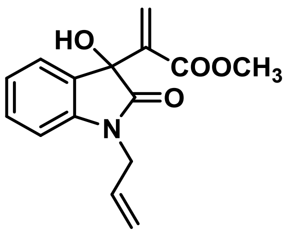

^{1}H NMR (400 MHz, DMSO-*d6*): δ 2.25 (*s*, 3H, CH_3), 3.05 (*m*, 2H, =CH_2), 3.96 (*dd*, *J* = 4,0 Hz, *J* = 8.0 Hz, 2H,CH_2), 4.60 (*m*, 1H, =CH), 5.27 (*dd*, *J* = 4,0 Hz, *J* = 8,0 Hz, 2H, =CH_2), 5,46, (*s*, 1H, OH), 5.71 (*m*, 2H, Ar–H), 5.80 (*dd*, *J* = 4,0 Hz, *J* = 4,0 Hz, 1H, Ar–H), 6.03 (td, *J* = 4,0 Hz, *J* = 4,0 Hz, *J* = 12,0 Hz, 1H, Ar–H). ^{13}C NMR (100 MHz, DMSO-*d6*): δ 41.7, 51.9, 74.9, 109.3, 117.2, 122.1, 123.1, 127.7, 129.5, 130.9, 143.7, 164.5, 175.3.

Methyl 2-(1-benzyl-3-hydroxy-2-oxoindolin-3-yl)acrylate (2c):

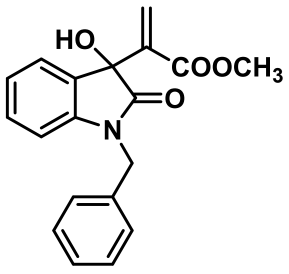

^{1}H NMR (500 MHz, $CDCl_3$): δ 7.20 (m, 5H), 7.17 (m, 2H), 6.98 (m, 1H), 6.71 (d, *J* = 9.6 Hz, 1H), 6.59 (s, 1H), 6.45 (s, 1H), 4.87 (m, 2H), 3.59 (s, 3H). ^{13}C NMR (125 MHz,

$CDCl_3$): δ 176.6, 165.2, 143.8, 139.2, 135.6, 130.3, 129.5, 128.9, 128.1, 127.8, 127.5, 124.0, 123.1, 109.9, 76.4, 52.2, 44.3.

Methyl 2-(5-chloro-3-hydroxy-1-methyl-2-oxoindolin-3-yl)acrylate (2d):

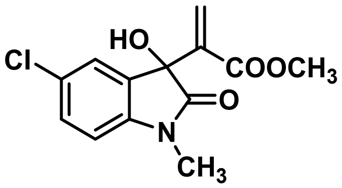

^{1}H NMR (400 MHz, $CDCl_3$): δ 7.30 (d, J = 7,6 Hz, 1H), 7.16 (d, J = 7,4 Hz, 1H), 7.01 (t, J = 7,5 Hz, 1H), 6.84 (d, J = 8,0 Hz, 1H), 6.55 (s,1H), 6.42 (s, 1H), 3.61 (s, 3H), 3.24 (s, 3H). ^{13}C NMR (100 MHz, $CDCl_3$)**:** δ 176.4, 165.2, 144.7, 139.3, 130.4, 129.4, 127.8, 124.0, 123.1, 108.8, 76.0, 52.2, 26.6.

Methyl 2-(5,7-dichloro-3-hydroxy-1-methyl-2-oxoindolin-3-yl)acrylate (2e):

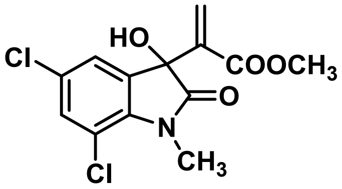

^{1}H NMR (400 MHz, DMSO-*d6*): δ 3.45 (*s*, 3H, CH_3), 3.55 (*s*, 3H, CH_3). 6.54 (*d*, J = 16,0 Hz, 2H, =CH_2), 7.00 (*s*, 1H, Ar–H), 7.05 (*s*, 1H, Ar–H), 7.49 (*s*, 1H, OH). ^{13}C NMR (100 MHz, DMSO-*d6*): δ 29.3, 52.1, 74.3, 114.9, 122.5, 126.7, 128.8, 130.6, 135.5, 138.4, 139.3, 164.4, 175.5.

Methyl 2-(1-allyl-3-hydroxy-5-methyl-2-oxoindolin-3-yl)acrylate (2f)

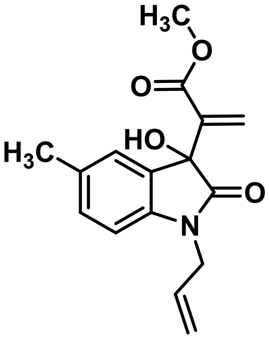

^{1}H NMR (400 MHz, DMSO-*d6*): δ 2.21 (s, 3H), 4.18 – 4.30 (m, 2H), 5.16 – 5.19 (d, J = 1.72 Hz, J = 10.4 Hz, 1H; 5.33 – 5.37(d, J = 1.72 Hz, J = 17.3 Hz, 1H), 5.78 – 5.87 (m, 1H); 6.47 – 6.50 (dd J = 1.32 Hz, J = 11.6 Hz, 2 H); 6.60 (s, 1H); 6.77 – 6.79 (d, J = 7.92

Hz, 1H), 6.85 (s, 1H), 7.05 – 7.07 (d, *J* = 8.8 Hz, 1H). ^{13}C NMR (100 MHz, DMSO-*d6*): δ 20.6, 41.8, 75.1, 109.1, 117.1, 123.8, 127.7, 129.6, 131.0, 131.1, 132.1, 139.8, 141.3, 164.6, 175.3.

Methyl 2-(5-fluoro-3-hydroxy-1-methyl-2-oxoindolin-3-yl)acrylate (2g)

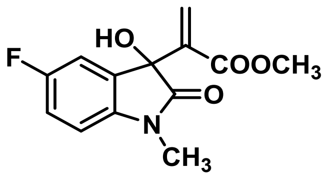

^{1}H NMR (400 MHz, DMSO-*d6*): δ 3.12 (*s*,3H, CH_3), 3.50 (*s*, 3H, CH_3), 6.47 (*d*, *J* = 1,20 Hz, 1H =CH_2), 6.50 (*d*, *J* = 1,12 Hz, 1H, =CH_2), 6.75 (*s*, 1H, OH), 6.87 – 6.90 (*dd*, *J* = 2,6 Hz, *J* = 7,8, 1H, Ar–H), 6.98 – 7.01 (*dd*, *J* = 8,6 Hz, *J* = 4,3 Hz, 1H, Ar–H), 7.11 – 7.16 (*td*, *J* = 8,4 Hz, *J* = 2,8 Hz, 1H, Ar–H). ^{13}C NMR (100 MHz, DMSO-*d6*): δ 26.3, 51.8, 75.1, 109.4, 111.0, 115.5, 120.0, 132.7, 139.2, 140.8, 158.3, 164.4, 175.3.

Methyl 2-(1-allyl-5-fluoro-3-hydroxy-2-oxoindolin-3-yl)acrylate (2h)

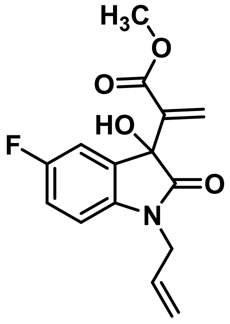

^{1}H NMR (400 MHz, DMSO-*d6*): δ 3.51 (*s*, 3H), 4.22 – 4.34 (qd, *J* = 4,8 Hz, *J* = 16,5 Hz, 2H), 5.18 – 5.21 (*dd*, *J* = 1,6 Hz, *J* = 10,4 Hz, 1H), 5.34 – 5.39 (*dd*, *J* = 1,6 Hz, *J* = 17,2 Hz, 1H), 5.77 – 5.87 (*m*, *J* = 5,16 Hz, *J* = 6,9 Hz, 1H), 6.49 – 6.55 (dd, *J* = 1,8 Hz, *J* = 15,6 Hz, 2H), 6.88 – 6.92 (m, 2H), 7.08 – 7.13 (td, J = 2,68 Hz, J = 8,6 Hz, 1H). ^{13}C NMR (100 MHz, DMSO-*d6*): δ 41.8, 51.9, 75.0, 110.1, 111.1, 115.5, 117.3, 128.3, 131.8, 132.7, 139.0, 139.9, 158.3, 164.5, 175.1.

Methyl 2-(1-benzyl-5-fluoro-3-hydroxy-2-oxoindolin-3-yl)acrylate (2i)

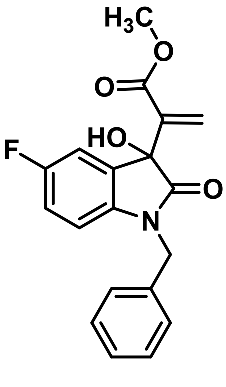

^{1}H NMR (400 MHz, DMSO-*d6*): δ 3.47 (*s*, 3H), 4.88 (s, 2H), 6.53 (d, *J* = 1,2 Hz, 1H), 6.57 (d, *J* = 1,2 Hz, 1H), 6.80 – 6.83 (dd, *J* = 4,3 Hz, *J* = 8,7 Hz, 1H), 6.91 (d, *J* = 2,7 Hz, 1H), 7.03 – 7.08 (td, *J* = 7,5 Hz, 1H), 7.33 – 7.36 (t, *J* = 7,4 Hz, 2H), 7.42 – 7.44 (d, *J* = 6,9 Hz, 2H). ^{13}C NMR (100 MHz, DMSO-*d6*): δ 43.1, 51.8, 75.1, 110.1, 111.1, 115.4, 117.3, 127.4, 128.4, 128.5, 132.7, 136.2, 138.9, 139.8, 158.3, 164.5, 175.4.

^1H and ^{13}C NMR spectra of 2a-2i

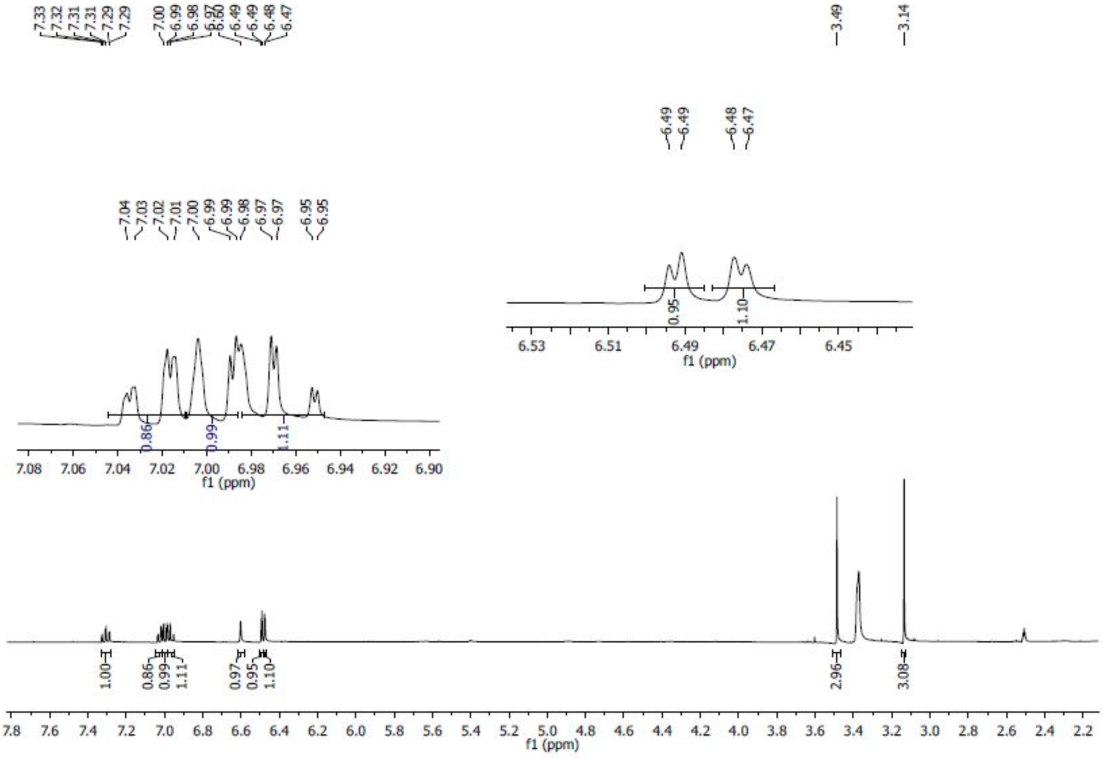

Figure S1: ^1H NMR spectrum of 2a.

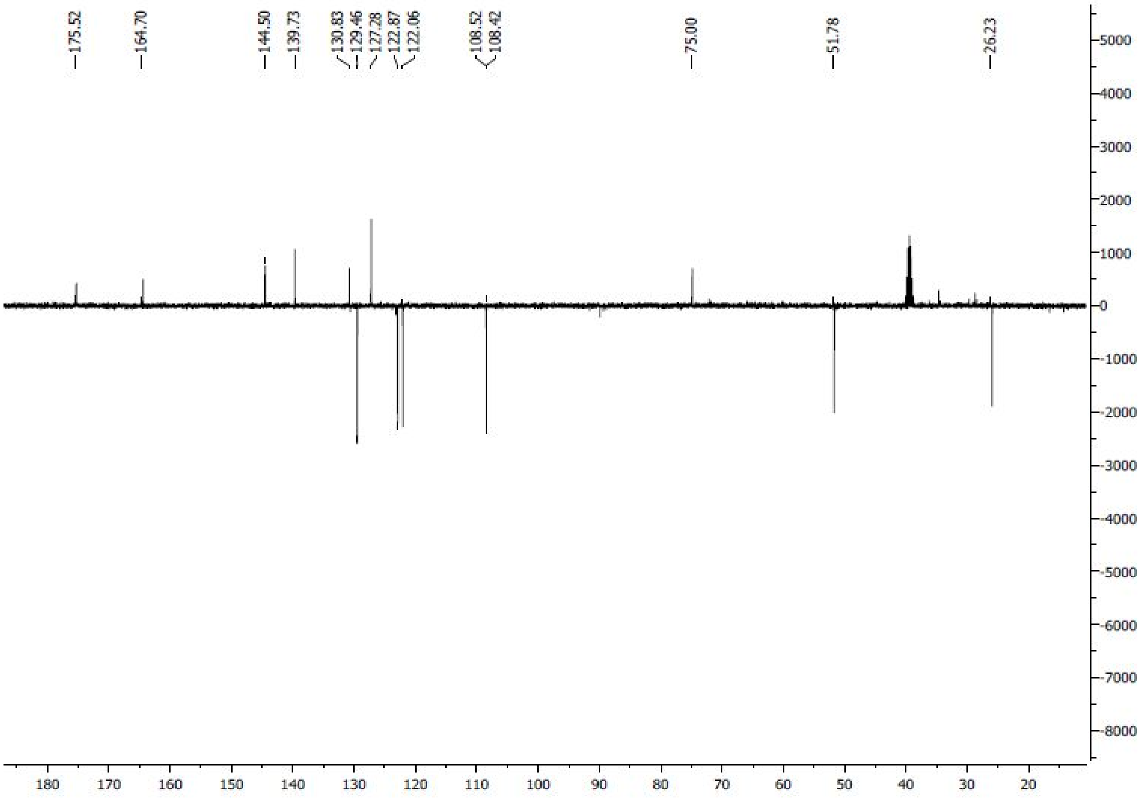

Figure S2: ^{13}C NMR spectrum of 2a.

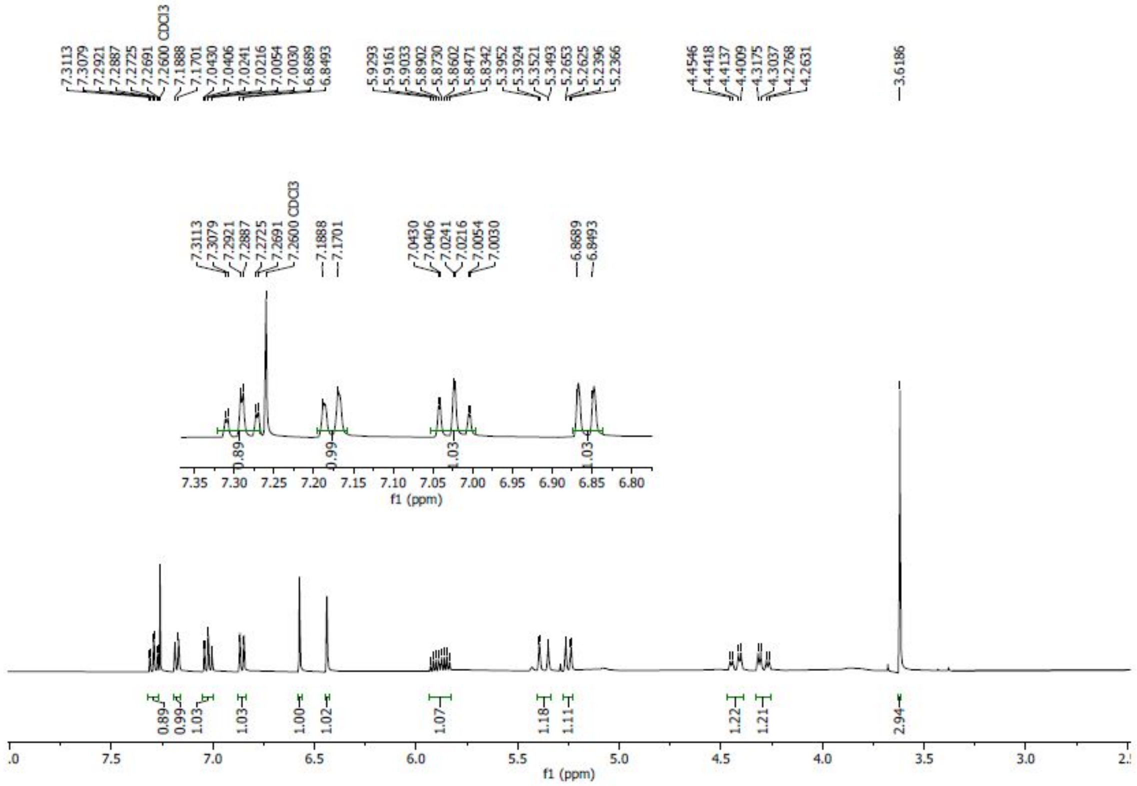

Figure S3: ^{1}H NMR spectrum of 2b.

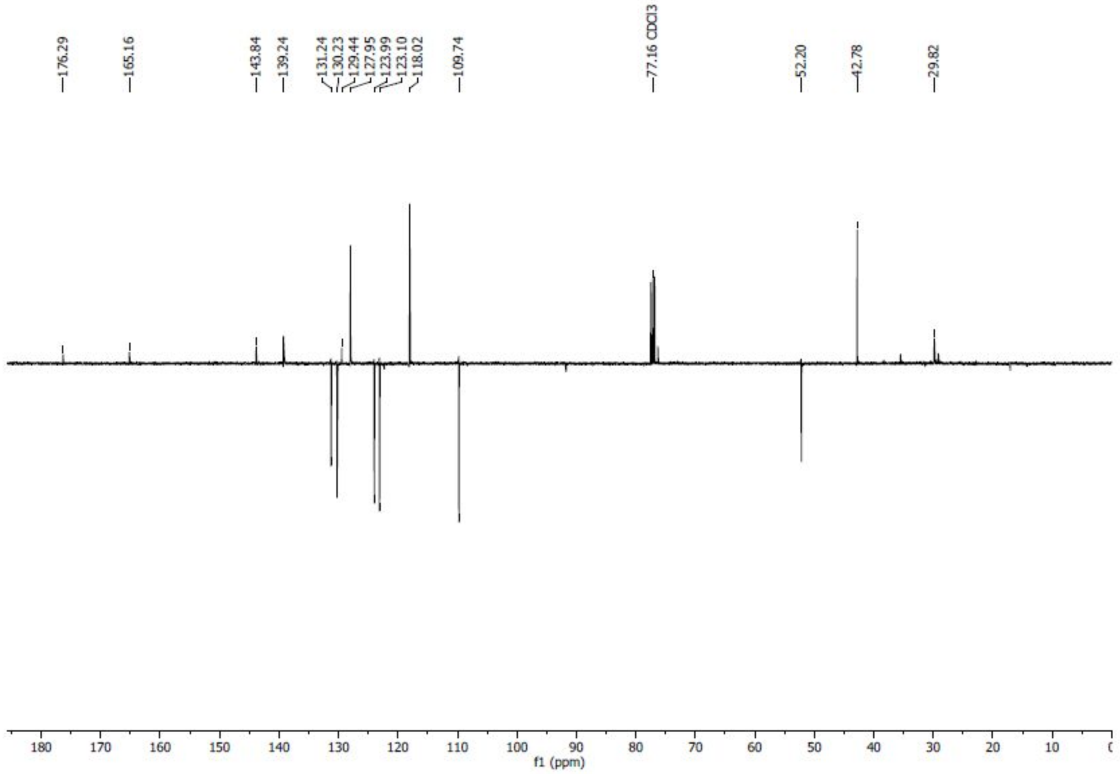

Figure S4: ^{13}C NMR spectrum of 2b.

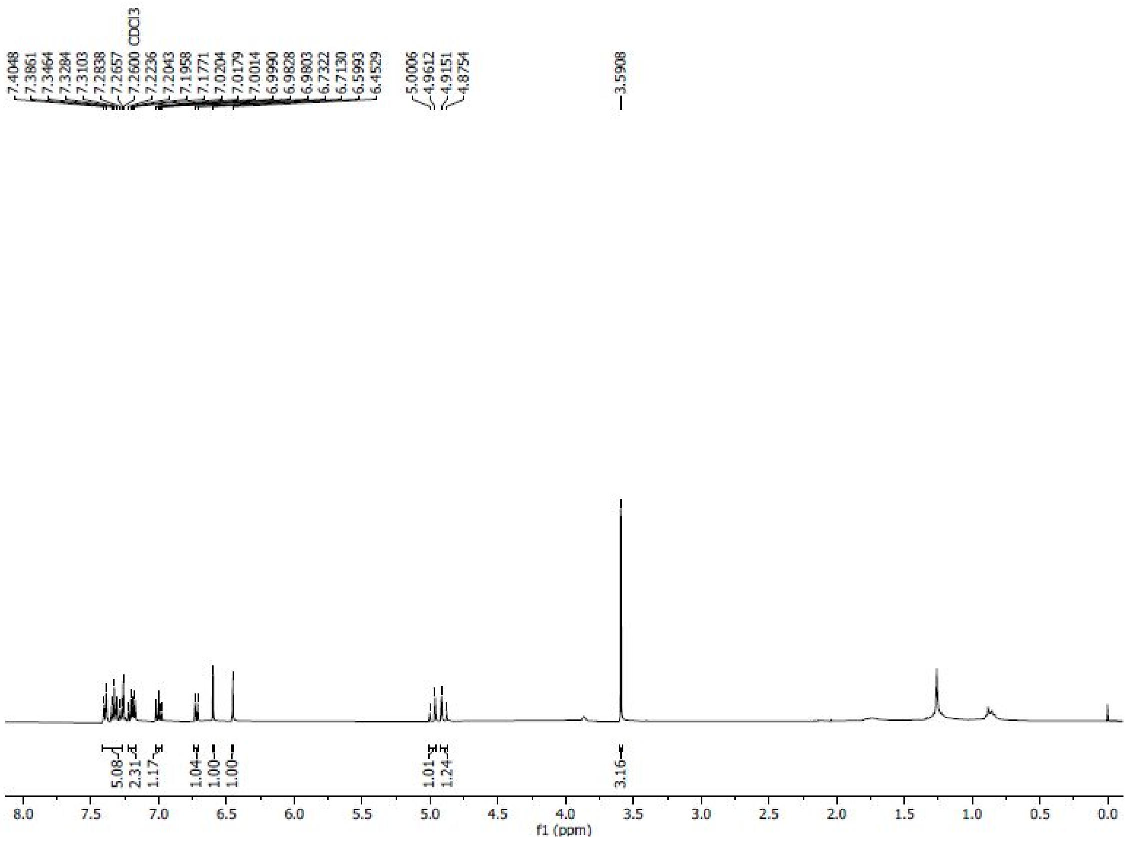

Figure S5: ^{1}H NMR spectrum of 2c.

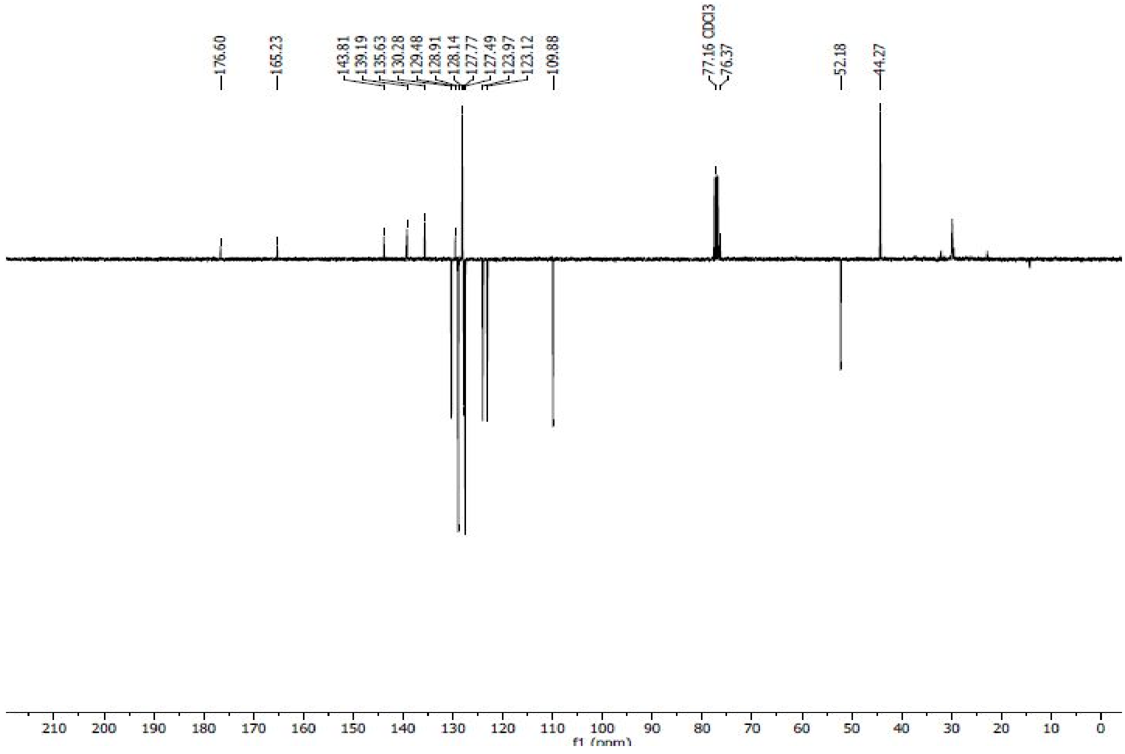

Figure S6: ^{13}C NMR spectrum of 2c.

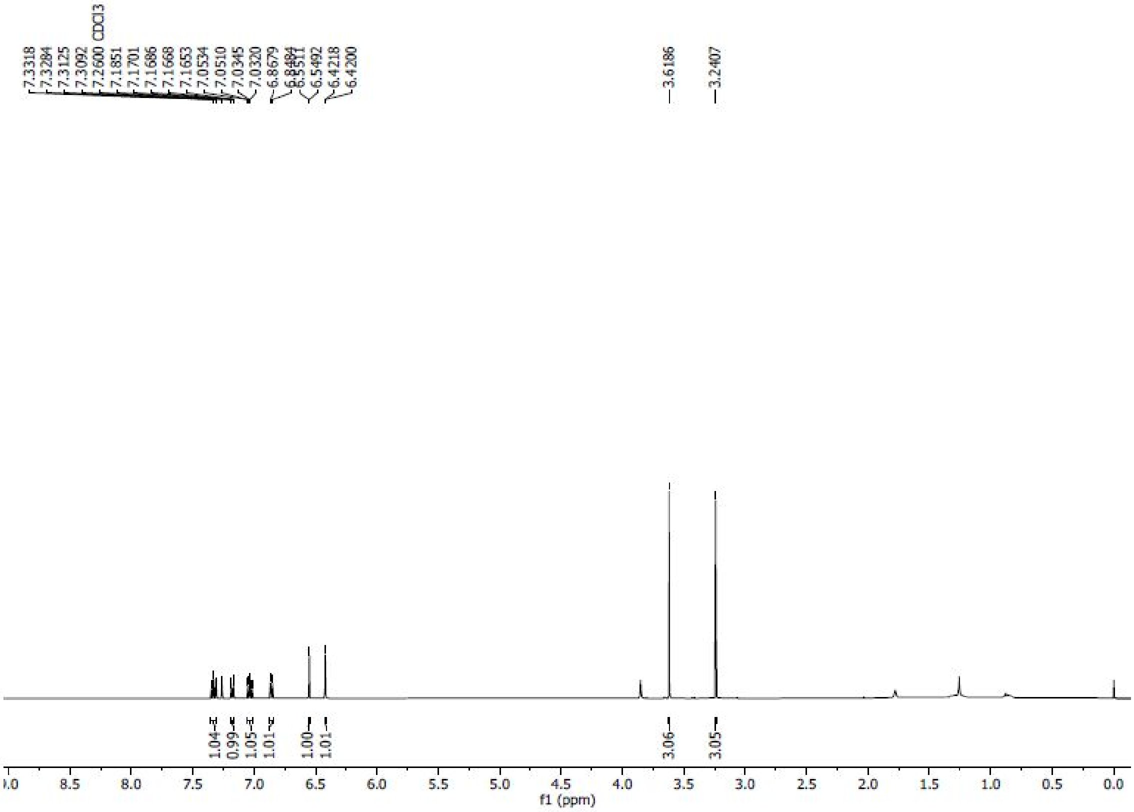

Figure S7: ^{1}H NMR spectrum of 2d.

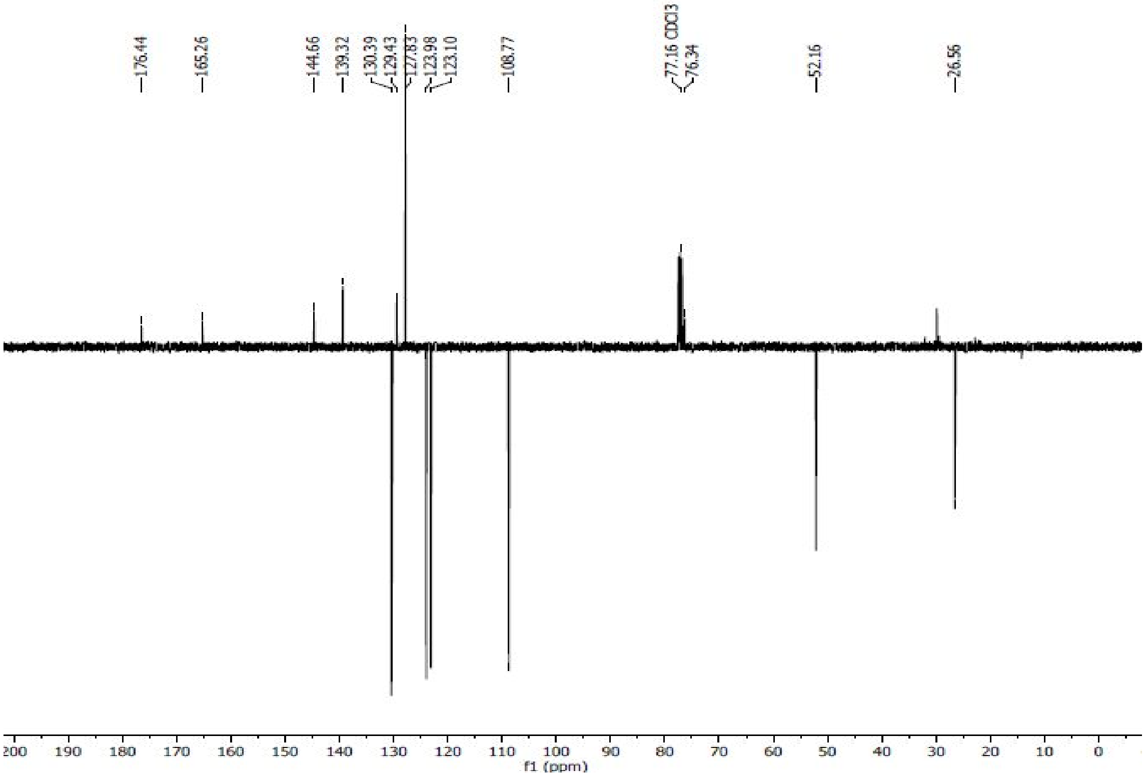

Figure S8: ^{13}C NMR spectrum of 2d.

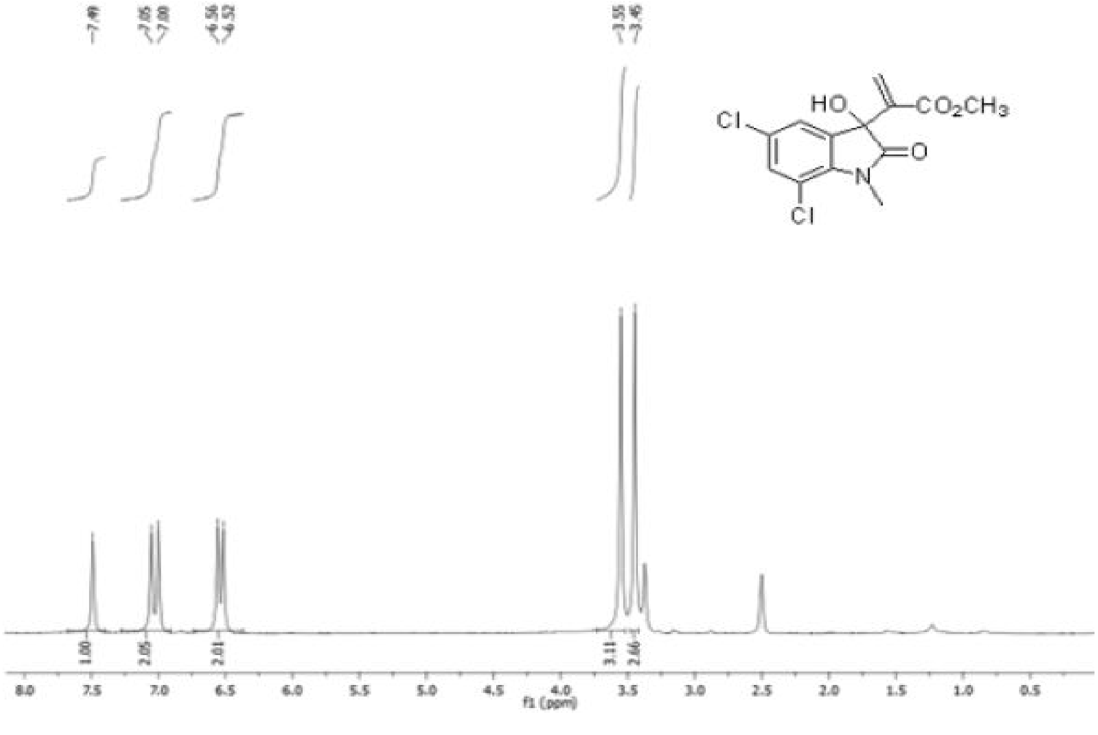

Figure S9: ^{1}H NMR spectrum of 2e.

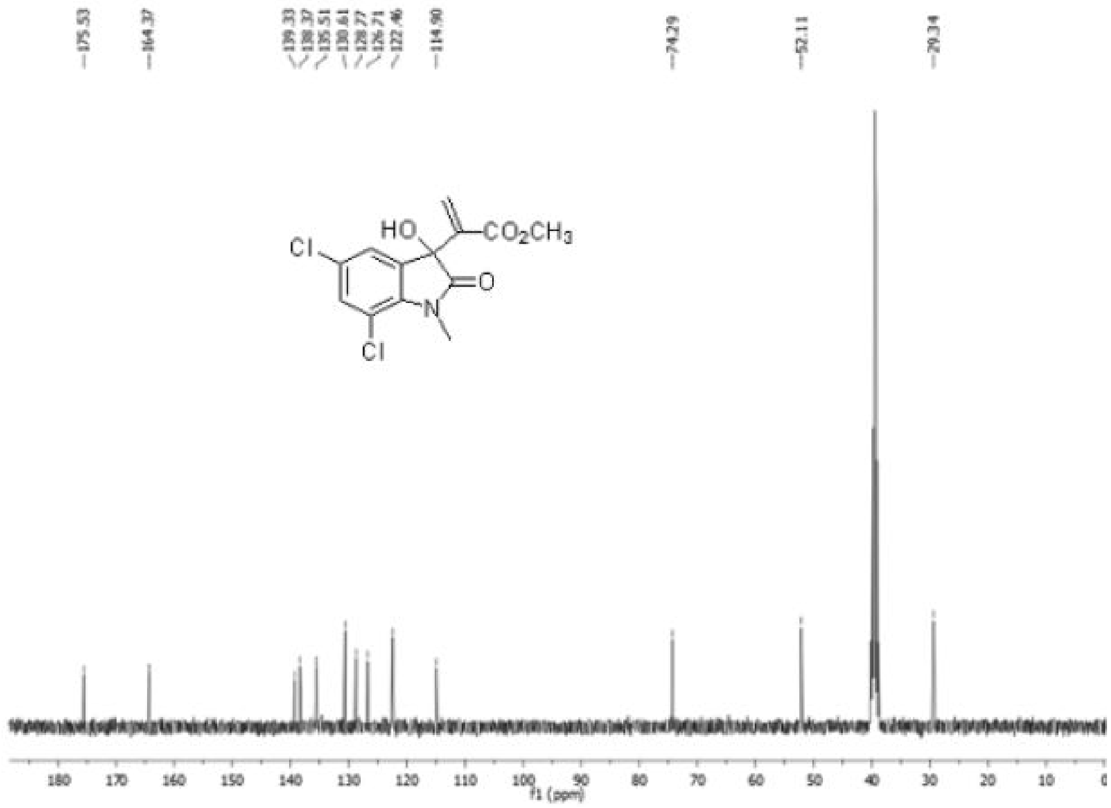

Figure S10: ¹³C NMR spectrum of 2e.

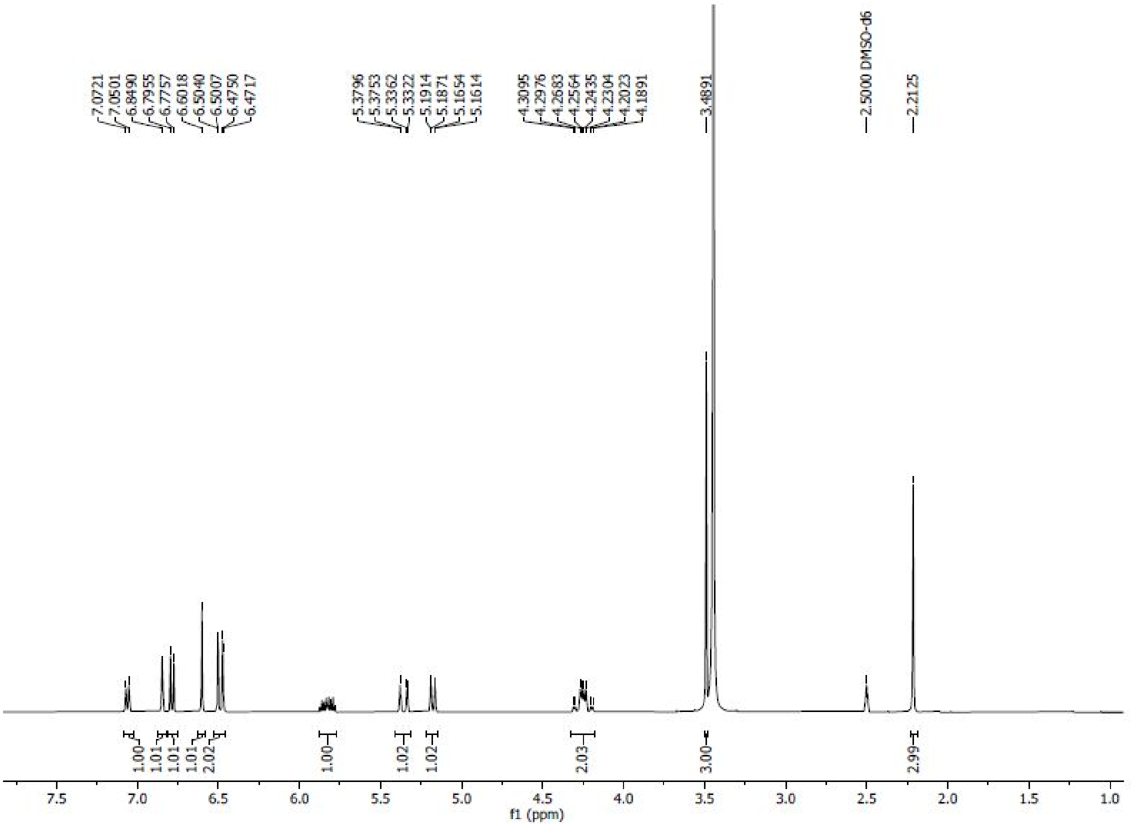

Figure S11: ¹H NMR spectrum of 2f.

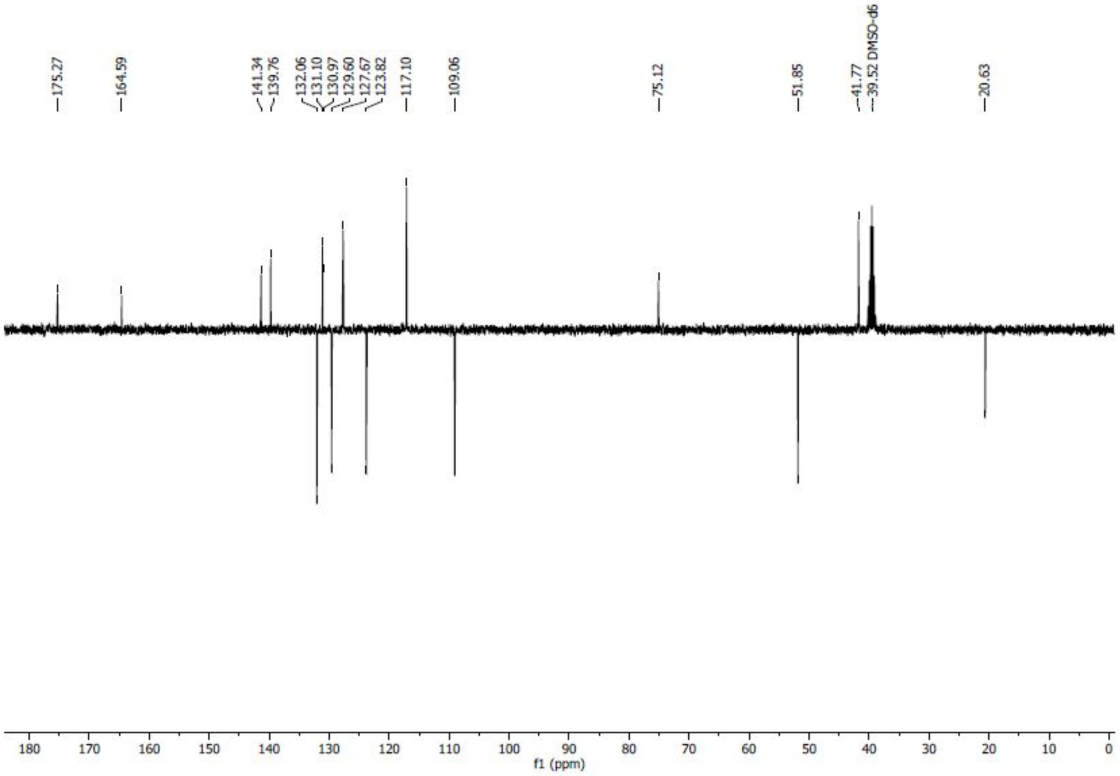

Figure S12: [12]C NMR spectrum of 2f.

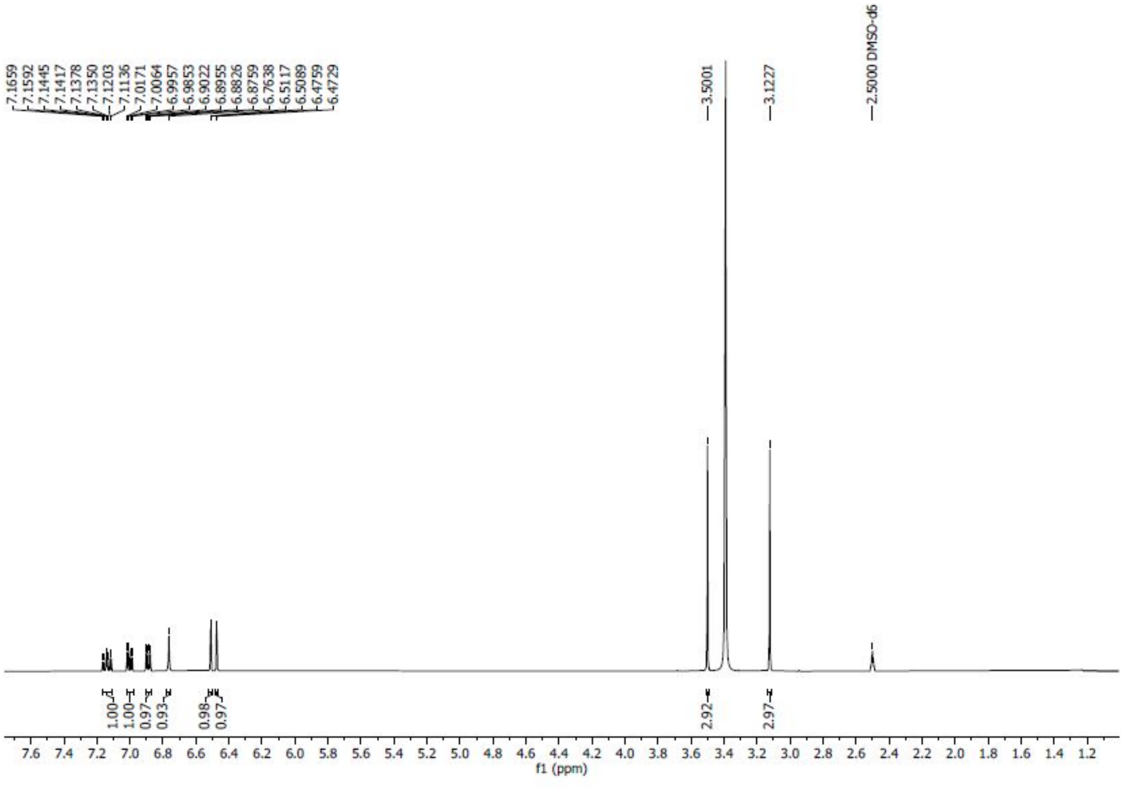

Figure S13: ^{1}H NMR spectrum of 2g.

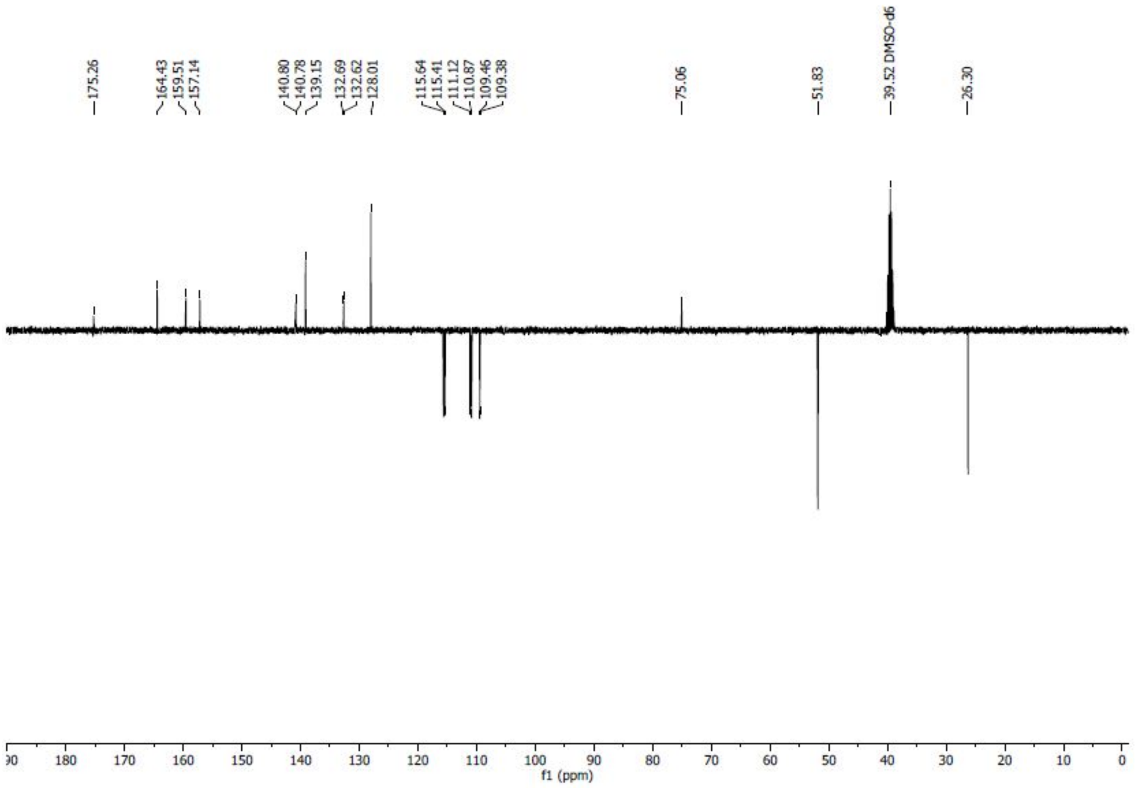

Figure S14: ^{13}C NMR spectrum of 2g.

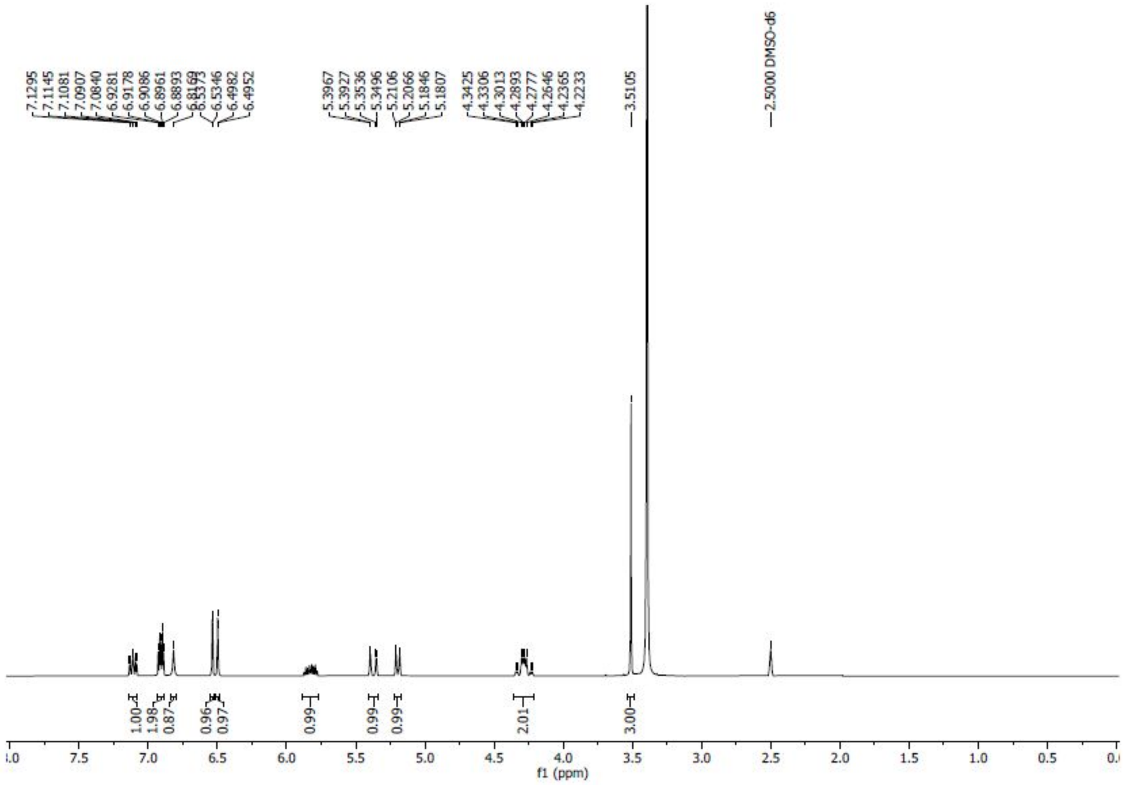

Figure S15: ^{1}H NMR spectrum of 2h.

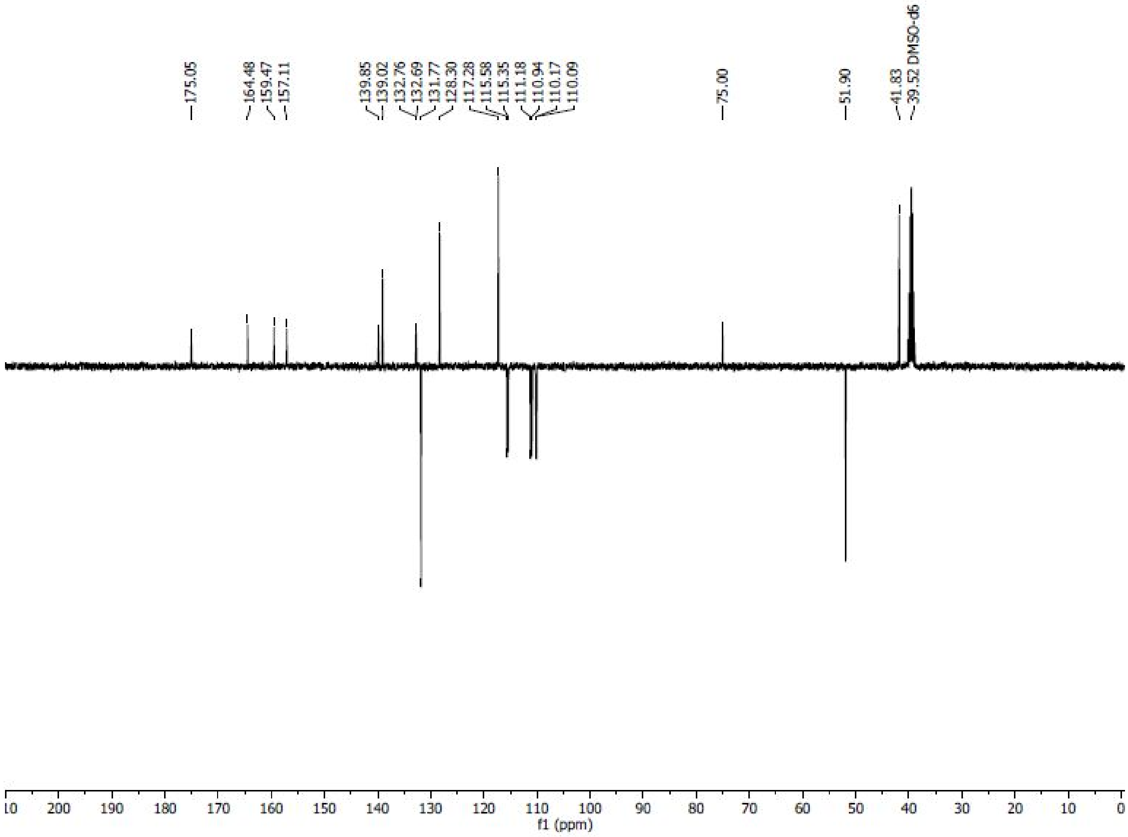

Figure S16: ^{13}C NMR spectrum of 2h.

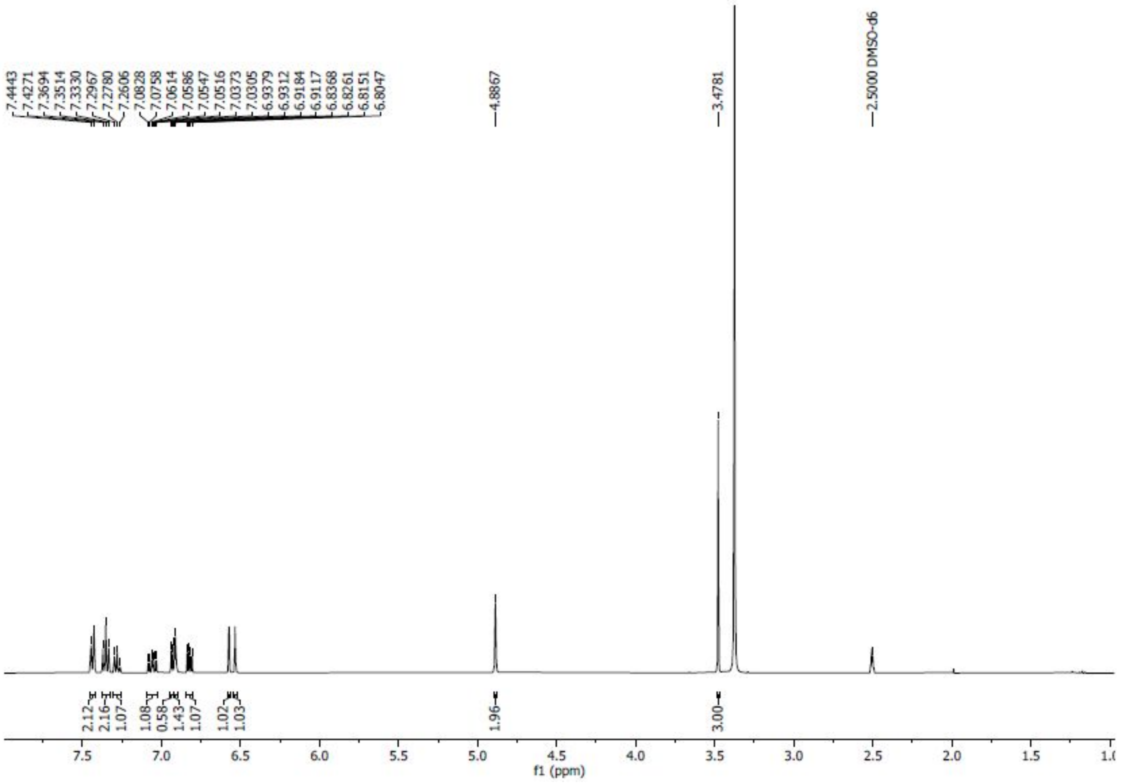

Figure S17: ^{1}H NMR spectrum of 2i.

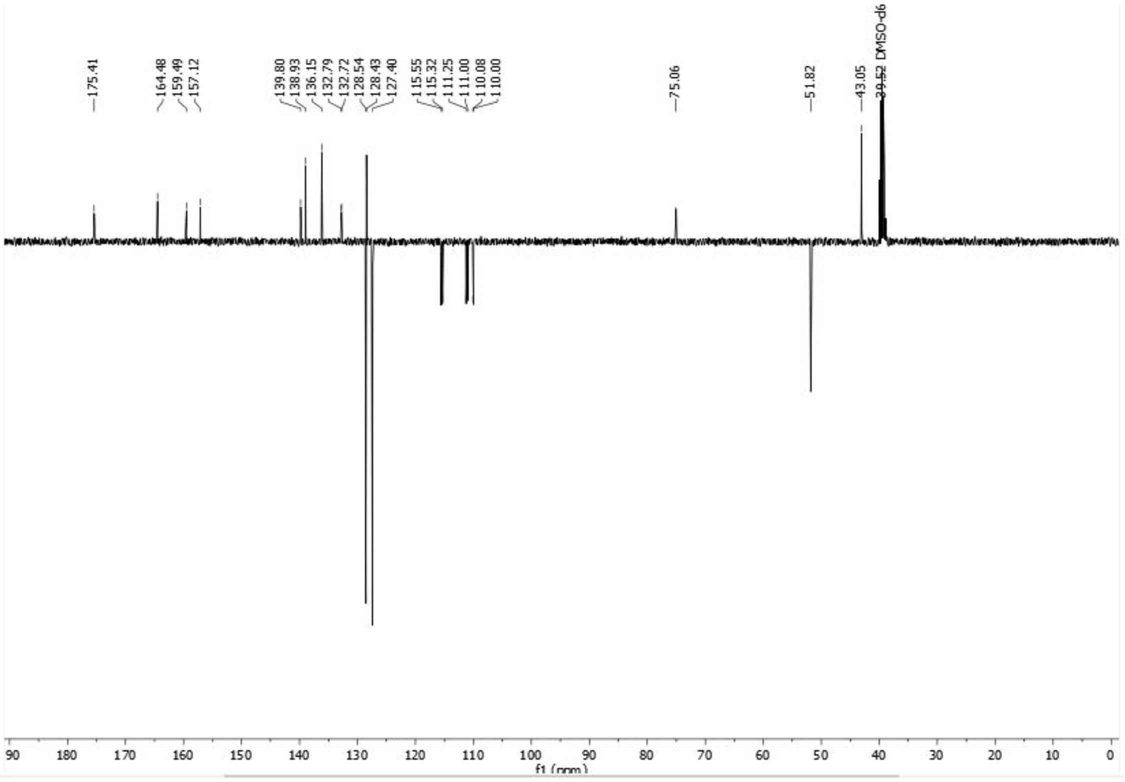

Figure S18: ^{13}C NMR spectrum of 2i.

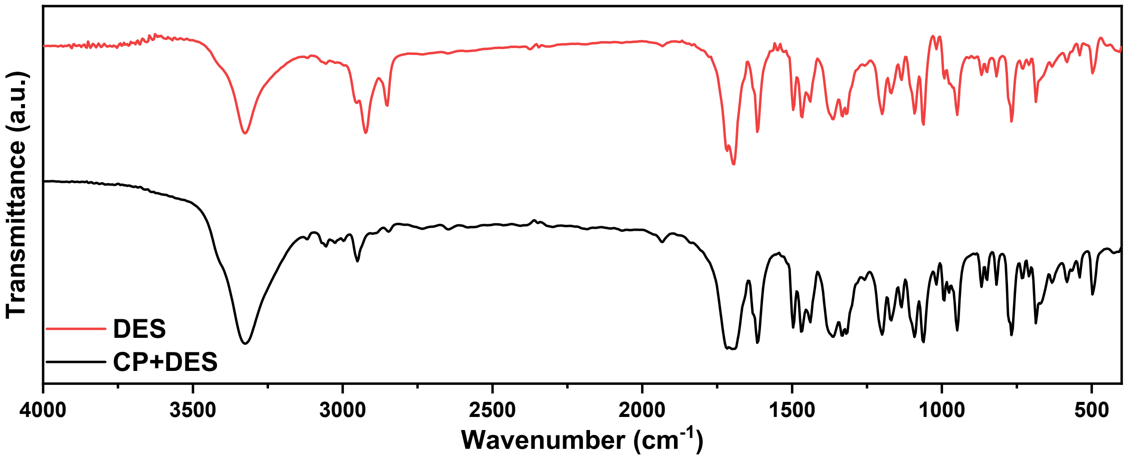

Figure S19: Infrared spectra for the 2g MBHA, synthesized in the presence and absence of the CP catalyst.
